# A Ligand-Directed Nitrophenol Carbonate for Transient in situ Bioconjugation and Drug Delivery

**DOI:** 10.1002/cmdc.202000655

**Published:** 2020-10-08

**Authors:** Anthony J. Burt, Parvaneh Ahmadvand, Larissa K. Opp, Austin T. Ryan, ChulHee Kang, Rock J. Mancini

**Affiliations:** [a] Department of Chemistry, Washington State University, NE College Ave, Pullman, WA 99164 (USA); [b] The Gene & Linda Voiland School of Chemical Engineering and Bioengineering, Washington State University, NE College Ave, Pullman WA 99164 (USA)

**Keywords:** Affinity Labelling, Avidin, Bioconjugation, Immunochemistry, Prodrugs

## Abstract

Here we report the first use of lig and-directed proximity accelerated bioconjug ation chemistry in the tandem delivery and release of a therapeutic payload. To do this, we designed a nitrophenol carbonate for ligand-directed in situ bioconjugation of a prodrug payload to a protein. The transient nature of our conjug ation chemistry renders the protein a depot for time-dependent release of active drug following hydrolysis and self-immolation. In our model system, using an immunostimulant prodrug, biotin lig and, and avidin protein, we observe release of bioavailable immunostimulant both spectroscopically and with an immune cell line over 48 h. Avidin co-crystalized with the nitrophenolate directing group verified the binding pose of the lig and and offered insig ht into the mechanism of in situ bioconjugation. Overall, this scaffold warrants further investigation for the time-dependent delivery of therapeutics and use in protein lig and pairs beyond biotin and avidin used for this work.

Bioconjugates are an alluring therapeutic modality that, relative to small molecules, suffer drawbacks in production including high cost, poor batch reproducibility, and poor shelf life; all of which hinder the development of bioconjugates as drugs.^[1]^ Despite these challenges, antibody drug conjugates such as brentuximab vedotin and trastuzmab emtansine which have found clinical success and FDA approval (2011 and 2013 respectively) exemplify the benefits of bioconjugates including tissue specificity,^[2]^ synergistic effects of the biomolecule and drug,^[3,4]^ and increased biological half-life.^[5]^ These examples and benefits have pushed researchers to explore further methodologies in bioconjugate drug delivery. For example, in situ bioconjugates have been explored using ligand-directed small-molecule probes whereby ligand-protein complexation leads to covalent affinity-labeling resulting in a bioconjugate.^[6]^ We envisioned that if this concept could be expanded to develop an affinity labeling platform that yields in situ bioconjugates capable of time-dependent drug release, this could present a new way to bypass the drawbacks of producing bioconjugate drugs while still harnessing the therapeutic benefits by producing them in situ.

Ligand-directed affinity labeling techniques have been pioneered by Hamachi and coworkers.^[7]^ They have developed multiple electrophilic moieties over the past decade capable of labeling nucleophilic amino acid side chains on proteins with small-molecule probes peripheral to the ligand binding site.^[8–16]^ It is hypothesized that upon ligand binding, an electrophilic moiety on the ligand-directed reagent is attacked by a nucleophilic side chain, leading to covalent attachment of the probe to the target protein. Ligand-directed affinity labeling has demonstrated success with soluble proteins such as carbonic anhydrase as well as membrane bound proteins such as the folate receptor with examples of in situ, in vitro, ex vivo, and in vivo labeling.^[11,17]^ Building on these concepts, we designed a scaffold (Figure 1) to allow for time-dependent drug delivery via a *transient* in situ bioconjugate formed by ligand-directed affinity labeling.

Our ligand-directed nitrophenol carbonate (LDNPC) chemistry was built around the leaving group ability of nitrophenols paired with the modest stability of nitrophenol carbonates at physiological conditions. To adapt the affinity probe to drug delivery, we included a self-immolative spacer^[18]^ between the carbonate electrophile and drug payload. We hypothesized that following affinity labeling of the payload and spacer to the target protein, hydrolysis would lead to self-immolation to liberate the active payload. Importantly, this scaffold can be built from inexpensive abundant precursors: *p*-nitrophenol, formaldehyde, cyanide, and ethylene glycol. LDNPC reagents exhibit excellent bench stability as a lyophilized powder and modest solution stability. Thus, we envision LDNPC reagents could mitigate many drawbacks of conventional bioconjugate drug production while retaining their alluring benefits via in situ bioconjugate synthesis.

For this proof-of-concept work, the protein ligand pair of avidin-biotin was chosen because: 1) the avidin-biotin crystal structure is known, and verified the presence of possible peripheral nucleophiles (Nu) to the biotin binding site.^[19]^ 2) The high affinity of biotin for avidin (*K*_d_ ≈ 10^−15^ M)^[20]^ was hypothesized to lead to rapid covalent labeling. 3) We believe that LDNPC chemistry could add a new tool to the many established applications that utilize avidin-biotin biotechnology.

The imidazoquinoline immunostimulant Imiquimod (IMQ)^[21–24]^ was chosen as payload for our proof-of-concept LDNPC reagent because our previous work with enzyme-directed IMQ allowed us to estimate its behavior as a prodrug.^[25,26]^ Based on this, and the well-defined structure activity relationship of the imidazoquinoline drug class,^[27,28]^ we hypothesized that linkage at the aminoquinoline nitrogen would lead to abrogated immunostimulatory activity and therefore allow the LDNPC reagent, as well as the covalently labeled avidin complex to serve as a prodrug of IMQ. Furthermore, the IMQ payload is an active immunostimulant for the RAW-Blue murine macrophage cell line (RB Cells) which links activation of the inflammatory transcription factor NF-*k*B to secreted embryonic alkaline phosphatase which can be readily quantified by colorimetric assay. For these reasons, the utility of LDNPC chemistry as a drug delivery technique was demonstrated using a biotin directing group to render avidin as a depot for time-dependent IMQ release in situ.

The biotin-LDNPC-IMQ reagent (**8**) was synthesized via the following convergent synthetic approach (Figure 2a) in which ligand (Fragment **A**) can be combined with nitrophenol moiety (Fragment **B**) and the resulting biotin-phenol (Fragment **A**+**B**) is then attached to IMQ payload (Fragment **C**) to yield LDNPC (**8**). Fragment **A** is prepared in one step by the known procedure of activating the carboxylate of biotin as succinimidyl ester (**1**) with EDC.^[29]^ Fragment **B** is prepared over three steps from *p*-nitrophenol (PNP). Briefly, chloromethylation of PNP is accomplished under acidic conditions with methylal and HCl gas.^[30]^ The resultant benzyl chloride (**2**) is displaced by cyanide to yield benzyl cyanide (**3**) which is subsequently reduced with borane to yield primary amine (**4**).^[31]^ Fragment **C** is synthesized over two steps from ethylene glycol and *p*-nitrophenyl chloroformate, yielding the ethylene glycol dicarbonate (**5**). Symmetry of (**5**) is broken under microwave conditions with IMQ to yield (**6**), the intermediate self-immolative spacer (Fragment **C**). Fragments **A** and **B** are combined in DMF with tertiary amine base to yield biotin-phenol (**7**), and then reacted with Fragment **C** under basic conditions. After 24 h, equilibrium is reached and LDNPC (**8**) is obtained in 8.7% yield over 5 synthetic steps from PNP. Complete synthetic procedures and characterization may be found in the Supporting Information.

With LDNPC (**8**) in hand, we next characterized aqueous stability. First, the p*K*_a_ of biotin-phenol leaving group (**7**) was determined to be 8.02 by titration with sodium hydroxide in water (Figure S1) suggesting that LDNPC (**8**) should be more resistant to base mediated hydrolysis at lower pH values. Aqueous stability of (**8**) was monitored over 48 h at 37°C in citrate phosphate buffer at pH: 4, 5, 6, and 7, typical of cellular organelles,^[32]^ and release of IMQ from direct hydrolysis was quantified by HPLC. Here, LDNPC (**8**) displayed a trend of increasing aqueous stability with decreasing pH. However, even at pH 7, total IMQ released from direct hydrolysis over 4, 12, and 48 h was determined to be 0, 5, and 77%, respectively (Figure S2). Stability at pH 7 was adequate over 12 h considering the high, albeit attenuated, affinity of our LDNPC reagent for avidin. This is also similar to Hamachi et al. who observe saturated covalent labelling with their ligand-directed acyl imidazole chemistry within 7 h for carbonic anhydrase I.^[17]^

We next examined the influence of avidin on the degradation of LDNPC (**8**). Due to the 4-nitrophenolate leaving group of (**7**), it was observed that cleavage of the carbonate phenol bond liberates a colorimetric indicator of degradation. We exploited this property to follow degradation of (**8**) in pH 7 buffer, with avidin or bovine serum albumin (negative control). In the presence of avidin, significant (80 μM) release of (**7**) was measured compared to minimal hydrolytic degradation with BSA or buffer alone (*<*20 μM). Expectedly, competition from biotin attenuates degradation emphasising the requirement for ligand complexation and nucleophilic attack (Figure 2b, S3). After using colorimetry to observe the initial formation of bioconjugate over 90 min, we next used HPLC to quantify the release of IMQ over 48 h with and without avidin. Both solutions showed measurable amounts of IMQ release. Interestingly, the sample with avidin showed a significant reduction in IMQ released (Figure S4), and these measurements combined with the colorimetric differences lead us to conclude that avidin is successfully labelled by LDNPC (**8**) and that the transient in situ bioconjugate degrades more slowly than (**8**) alone. Based on the possible nucleophilic side chain addition to (**8**), we expect the resulting bioconjugate to be considerably more stable than the carbonic acid formed from direct hydrolysis. Therefore, although subsequent experiments were unable to determine the exact Nu that participates in conjugation, we reasoned that the results of this experiment are consistent with transient covalent attachment of the self-immolative spacer and IMQ to avidin.

After confirming that our LDNPC reagent could create an in situ bioconjugate with avidin, we turned to structural biology to gain insight into the possible residues acting as Nu. Due to hydrolysis of the bioconjugate over the crystallization period we were unable to obtain co-crystals of avidin with (**8**). We also attempted to crystalize avidin and soak in LDNPC (**8**), however, these crystals did not provide complete electron density maps. In lieu of this, we proceeded to co-crystalize avidin with the biotin-phenol leaving group (**7**). This approach resulted in a 1.58 Å resolution crystal structure of the avidin-biotin-phenol (**7**) complex (PDB: 6XND, Figure 3, Table S1).

As expected, the biotin moiety of (**7**) is situated in a similar binding pose as biotin,^[19]^ buried within the binding pocket of the eight-stranded anti-parallel β-barrel. Emerging from the pocket and surrounded by solvent accessible flexible loop regions is the nitrophenol moiety (Figure 3a). Isothermal titration calorimetry (ITC) shows the dissociation constant of the ligand to be *K*_d_=2.25 × 10^−7^ M (Figure S5), considerably weaker than biotin. We also determined conditions to directly compare the binding of (**7**) and (**8**) by ITC, and the results show binding affinities within one order of magnitude (Figure S6) in agreement with binding observed by HABA assay (Figure S7).

The nitrophenolate appears to be stabilized by hydrogen bonds from S101 and R114 to the phenol, as well as a Van der Waals interaction between A39 and the nitro group (Figure 3b). Possible Nu in the region (within 12 Å of phenolate) include T40, S41, S101, S102, T113 and K111. All are constituents of dynamic loop regions and therefore all potentially capable of interacting with LDNPC (**8**). Interestingly, the interface between the monomers of avidin position two equivalents of ligand within the same solvent accessible pocket, with the aromatic rings separated by only 8.4 Å in the static crystal structure (Figure 3c). This overcrowded region could also drive hydrolysis to release the payload thereby relieving steric strain and returning the protein to its native state. Taken together, this data concretely proves that the modified biotin reagent remains a ligand for avidin and that proximal Nu to the carbonate moiety of LDNPC (**8**) are abundant.

We next sought to demonstrate the prodrug nature of avidin labeled with LDNPC (**8**) and time-dependent release of IMQ from the resultant in situ bioconjugate. First, to compare the abrogated immunogenicity from LDNPC (**8**) to IMQ, we treated RB cells with equimolar concentrations (1–100 μM) of each respective compound (Figure S8). Because the RB assay itself is conducted over 16 h, we observed some immunogenicity resulting from expected hydrolysis of (**8**) in the cell media. Regardless, the results show abrogated activity of LDNPC reagent compared to IMQ, with significantly reduced immunogenicity for the LDNPC reagent at 50 and 100 μM (p*<*0.001). Even at lower concentrations (1, 5, and 10 μM) the trend of reduced immunogenicity is conserved. With proof of abrogated activity, we next designed an experiment to capture the time-dependent immunogenicity that results from release of IMQ while simultaneously highlighting that in situ bioconjugation results in ligand-directed immunogenicity. To accomplish these aims, we utilized a 10 kDa molecular weight cut off centrifugal filter device (CFD). The workflow of the assay (Figure 4a), begins by incubating avidin with LDNPC (**8**) for 60 s at 37°C to allow complexation. Next, the solution was spun through the CFD effectively isolating the IMQ-avidin bioconjugate. The bioconjugate was then washed, concentrated, reconstituted in buffer, and incubated for the indicated time (0–72 h at 12 h increments) before being used in a RB cell assay that was developed for 16 h (t=0 h was still incubated with RB cells for 16 h). The results of the assay show increased immunogenicity over time caused by transient bioconjugation of the LDNPC reagent to avidin. Any uncomplexed LDNPC reagent, and likewise any IMQ released by nonspecific hydrolysis (both <1 kDa) is washed through the CFD. Therefore, the observed immunogenicity in this assay results from in situ bioconjugation and subsequent hydrolysis from avidin. We anticipated that longer incubation times of the bioconjugate in buffer would result in greater IMQ release and therefore higher immunogenicity approaching the theoretical loading target of 50 μM. Indeed, increasing immunogenicity with increasing incubation time was observed, matching the 50 μM IMQ standard by 24 h (Figure 4b). Additionally, the avidin concentration required to deliver 50 μM of IMQ from the bioconjugate showed small but non-negligible immunogenicity on its own and we attribute this to the later time points exceeding the IMQ standards. Overall, this experiment highlighted the ability of the bioconjugate to release a biologically relevant substrate, in a time-dependent manner, sustained over several days after in situ labeling with LDNPC (**8**).

In conclusion, we demonstrated ligand-directed nitrophenol carbonate (LDNPC) chemistry in a proof-of-concept example that forms a transient in situ bioconjugate between avidin and a self-immolative spacer with imidazoquinoline payload. We have shown, using structural biology and ITC, that the biotin modified with nitrophenolate, remains a viable ligand (*K*_d_=2.25 × 10^−7^ M) for avidin. Although we have yet to conclusively prove if IMQ is conjugated through a carbamate or carbonate bond, the crystal structure of avidin with the biotin-phenol leaving group (**7**) demonstrates the abundance of nucleophiles proximal to the biotin binding site. With S101 and K111 the two most spatially rational leads for further investigation into the exact mechanism of LDNPC mediated bioconjugation to avidin and subsequent payload release. Regardless, at this time, we have demonstrated that labeling of the biomolecule occurs, and that once labeled, the resulting bioconjugate releases its payload in a time-dependent manner over 72 h. We believe that this biotindirected example of LDNPC chemistry could find use in the mature field of avidin-biotin biotechnology where it can add an additional dimension of capability by introducing time-dependent avidin-directed substrates. More generally, we have demonstrated that LDNPC chemistry is a viable technique for the formation of transient in situ bioconjugates that can release a payload. Our lab intends to explore further ligand-protein pairs as well as therapeutic and diagnostic payloads within this framework.

## Supplementary Material

Supp info

## Figures and Tables

**Figure 1.**
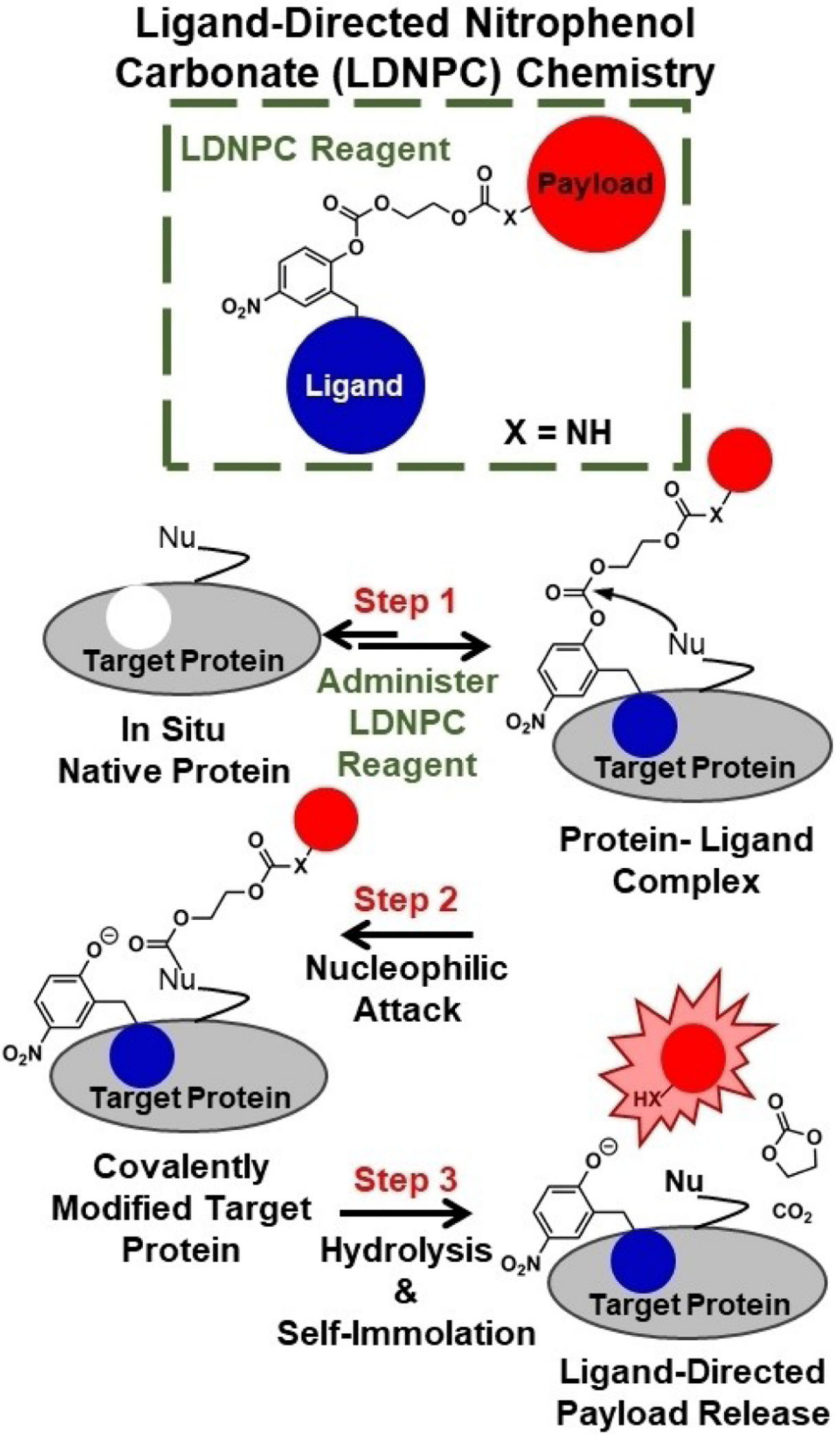
Drug delivery via ligand-directed nitrophenol carbonate (LDNPC) chemistry involves 3 steps to release active payload. **Step 1**. Addition of LDNPC reagent to target protein results in protein-ligand complex. **Step 2**. Nucleophile (Nu) peripheral to the binding site attacks the LDNPC reagent displacing the nitrophenolate-modified ligand, resulting in a covalently modified target protein. The nitrophenolate-modified ligand is free to dissociate. **Step 3**. Hydrolysis and self-immolation result in release of bioavailable payload at the location of the covalently modified protein. In this work X=NH.

**Figure 2.**
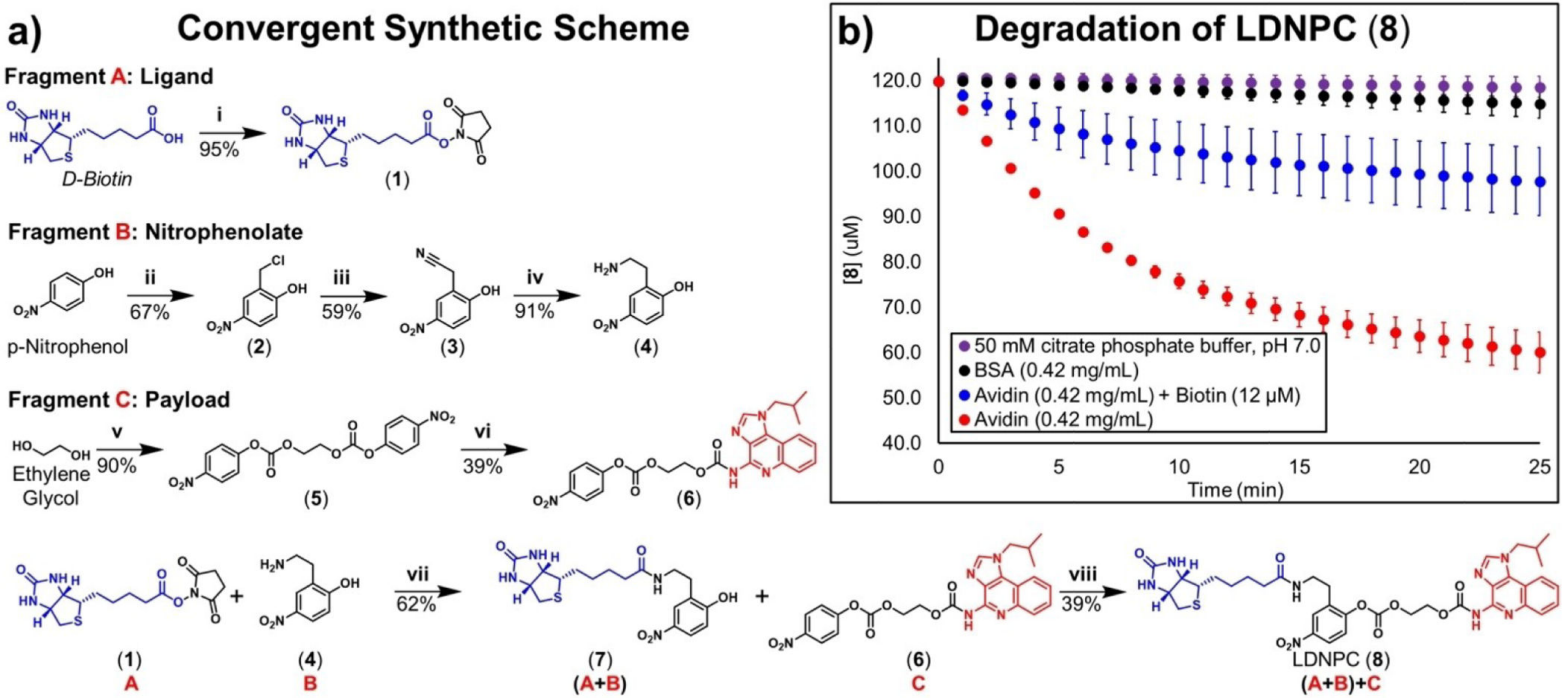
**a)** Synthetic route to ligand-directed nitrophenol carbonate (LDNPC) (**8**). **Fragment A i**: EDC-HCl, NHS, DMF, RT 24 h. **Fragment B ii**: Methylal, HCl (*g*), HCl *conc.*, H_2_SO_4_ *conc.*, 72°C, 4 h. **iii**: Acetonitrile, 5 M KCN (*aq*), 0°C 30 min, 60°C 30 min. **iv**: 1) BH_3_-THF 1 M in THF, 100°C, 4 h. 2) HCl 0.8 M in MeOH, 100°C, 12 h. **Fragment C v**: *p*-Nitrophenyl chloroformate, pyridine, DCM, 90°C, 24 h. **vi**: Imiquimod, THF, MW Irradiation: 90°C, 55 min, 1 bar. **(A**+**B) vii**: DIPEA, DMF, RT, 18 h. **(A**+**B)**+**C viii**: DIPEA, DMF, RT, 24 h **b)** Degradation kinetics of 120 μM LDNPC (**8**) was measured as liberated nitrophenolate (**7**) in the presence of Avidin (**Red**, 0.42 mg/mL), Avidin+Biotin (**Blue**, 0.42 mg/mL, 12 μM), Bovine Serum Albumin (BSA) (**Black**, 0.42 mg/mL) or citrate phosphate buffer (**Purple**, 50 mM pH 7). The effects of avidin on LDNPC (**8**) are significant compared to BSA and Buffer (p *<*0.05 calculated at 2, 5, 10, 15, 20 min; error bars are standard deviation of the mean for triplicate experiments). See the Supporting Information for longer timepoints.

**Figure 3.**
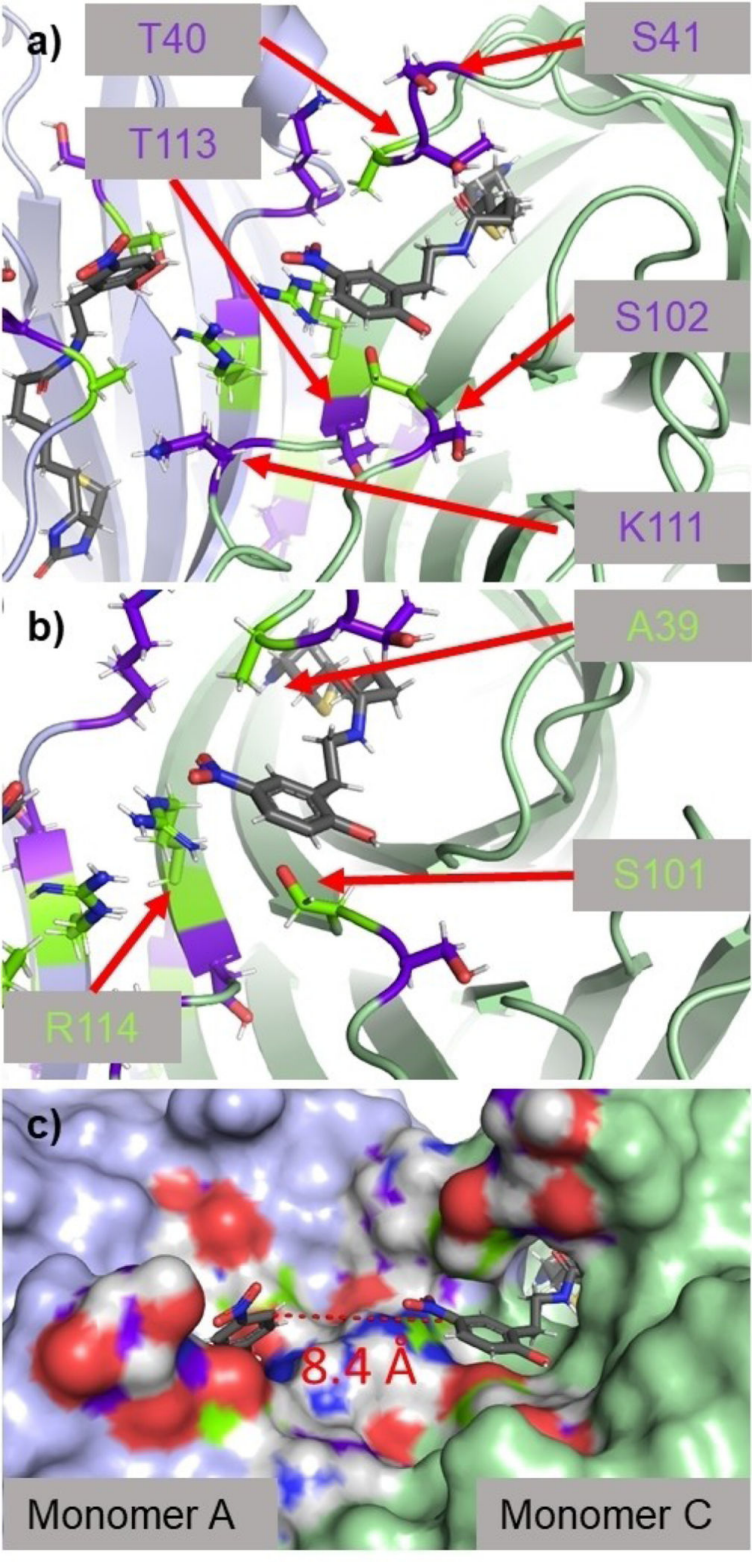
X-ray crystal structure (1.58 Å resolution) of biotin-phenol leaving group (**7**, grey) bound to avidin. **a)** Flexible loop regions around nitrophenolate moiety with proximal (*<*12 Å from phenolate) nucleophiles (Nu) on a single monomer unit shaded in purple including residues S41,102, T40,113, and K111. Monomer A and C are shaded lavender and light green, respectively. **b)** Highlight of residues A39, S101, R114 (lime green) directly implicated in stabilizing nitrophenolate in the avidin binding pocket. **c)** View showing electrostatic surface. Distance between ligand bound to monomer A and C is only 8.4 Å between the aryl rings. This view also shows how the nitrophenolate moiety is positioned just outside the binding pocket in a solvent accessible region.

**Figure 4.**
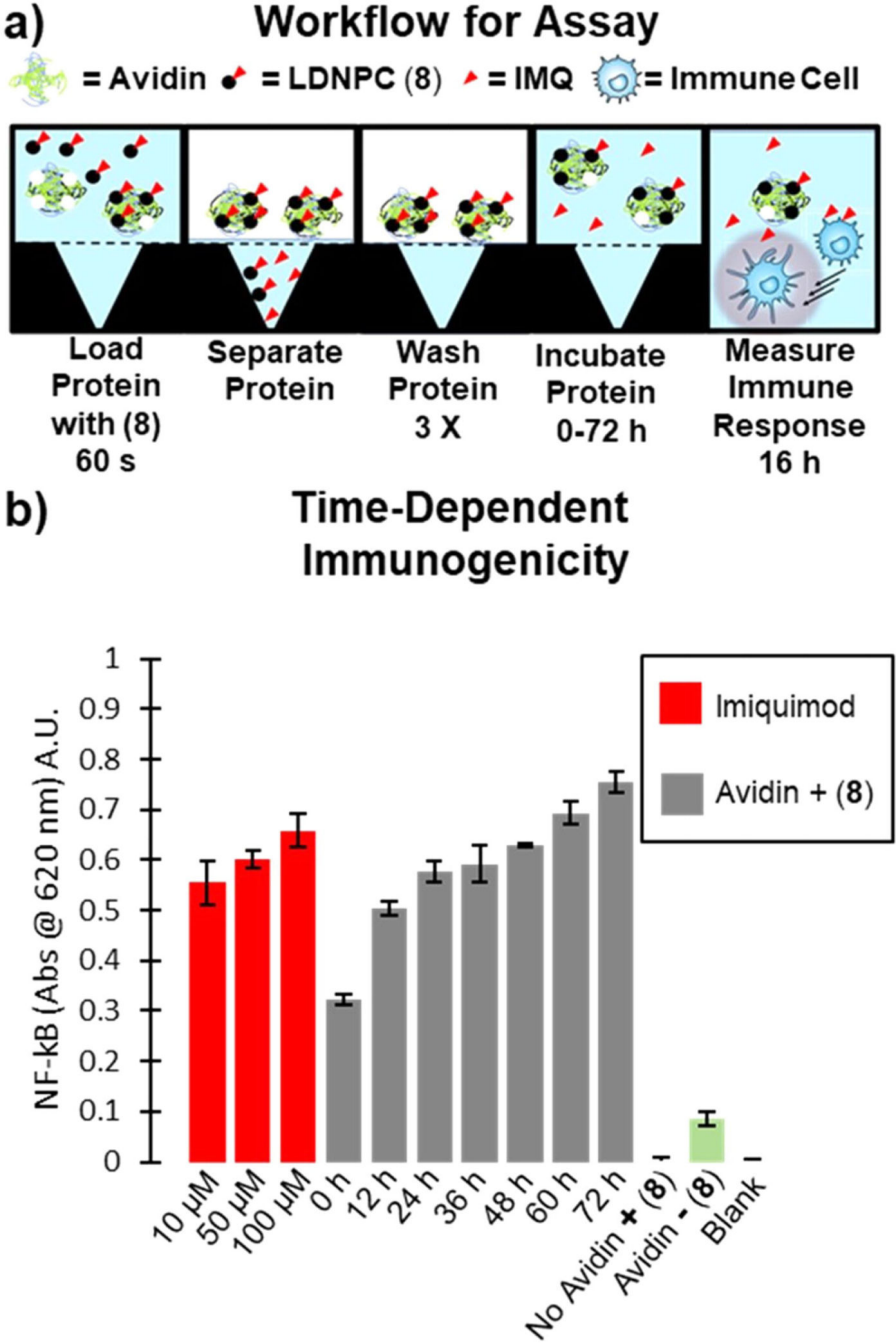
**a)** Workflow of time-dependent immunogenicity assay. Avidin was incubated with (**8**) for 60 s, at 37°C, before separation with 10 kDa MWCO centrifuge device (14,000 RCF, 5 min, 37°C). Avidin was washed (PBS 3×300 μL) and then incubated for indicated time prior to running a RB Cell assay to measure immunogenicity. **b)** The assay resulted in time-dependent immunogenicity from avidin loaded with (**8**) (**Grey**). Theoretical loading was 50 μM of IMQ (**Red**) with comparable immunogenicity observed from the bioconjugate following 12–24 h of incubation. Avidin has small, but significant, immunogenicity (p*<*0.001) on its own compared to blank. Error bars are standard deviation of the mean for experiments performed in triplicate.
