# Supplementary material for: A Ligand-Directed Nitrophenol Carbonate for Transient in situ Bioconjugation and Drug Delivery
Source: ChemMedChem. Author manuscript; Available in PMC 2021 Nov 4. (PMC7702144; doi:10.1002/cmdc.202000655)
Supplement: Supp info [file NIHMS1637900-supplement-Supp_info.pdf]

Table of Contents:

Materials and Methods 2
Synthetic Procedures 3
Titration Protocol 9
Hydrolysis Assay Protocol 9
Avidin Crystallization and Structure Determination Protocol 10
HABA Competition Assay Protocol 10
Isothermal Titration Calorimetry Protocol 10
Cell Culture Protocol 11
RAW-Blue Assay Protocol 11
Secreted Embryonic Alkaline Phosphatase Colorimetric Assay Protocol 11
Time-Dependent Immunogenicity Assay Protocol 11
Supporting Figures and Tables 13
Characterization Data 22
References 31

Materials and Methods:

Materials. Unless otherwise noted, all chemical reagents were purchased from Sigma-Aldrich (St. Louis, MO), Fisher Scientific (Pittsburgh, PA), or VWR (Radnor, PA), and used as received. All solvents were purchased from Fisher or VWR and dried over 3 Å molecular sieves at least 48 h prior to use. Imiquimod was purchased from eNovation Chemicals LLC (Bridgewater, NJ). Deuterated dimethyl sulfoxide and chloroform were purchased from Cambridge Isotope Laboratories (Andover, MA). The RAW-Blue cell line was purchased from InvivoGen (San Diego, CA), and passages 5-20 were used for all experiments. Dulbecco's Modified Eagle Media (DMEM) and Dulbecco's Phosphate Buffered Saline (DPBS) were purchased from Sigma-Aldrich. Heat-Inactivated Fetal Bovine Serum (HI-FBS, Seradigm grade) was purchased from VWR. Avidin from chicken egg whites was purchased from Santa Cruz Biotechnology (Dallas, TX). Nanosep 10K OMEGA centrifugal filter devices (10 kDa MWCO CFD) were purchased from Pall Corporation (Port Washington, NY)

Methods. ^{13}C, ^{1}H, HSQCAD, and symmetrized COSY NMR spectra were taken on a Varian 400 MHz NMR spectrometer; 5 mm OneProbe, 2-channel multi-tunable probe with auto tuning and z-axis pulse field gradients, equipped with 96 position autosampler or a Varian DD2 600 MHz NMR Spectrometer , 5 mm OneProbe, automatic tuning and 12 position auto sampler. NMR data was analyzed using MestreNova software. Absorbance for UV/VIS (200 -1000 nm) measurements and RAW-Blue enzymatic assays (620 nm) were measured on a Fisher accuSkan GO UV/Vis microplate spectrophotometer. Conversion of LDNPC (**8**) to Imiquimod was monitored via NF-κB transcription in RAW-Blue cells using 5-bromo-4-chloro-3-indolyl phosphate p-toluidine salt (BCIP). Column chromatography was performed on a Teledyne ISCO (Lincoln, NE) Combi Flash Rf+ PurIon purification system using Silicycle (Quebec, Canada) 230-400 mesh 40-63 µm Cartridges. Microwave synthesis was performed using a Biotage (Uppsala, Sweden) Initiator+ SP Wave Microwave Reactor. HPLC was performed on a Dionex UltiMate 3000 HPLC (Thermo Fisher Scientific, Waltham, MA) equipped with Chromeleon software version V6.80 SR14 and a C18 analytical column (Phenomenex (Torrance, CA) Synergi 4 µm Hydro- RP 80A, 75 x 4.6 mm) at a flow rate of 1.0 mL min^{-1}, and UV detection at 254 nm. Mobile phase consisted of A: HPLC grade Water with 0.1% trifluoroacetic acid, B: HPLC grade Acetonitrile 0.1%Trifluoroacetic acid. Cells were cultured in a Thermo Fisher Scientific Forma Series II 3110 Water Jacket CO_2 incubator (model 3110, 37 °C, 5% CO_2). All centrifugation steps were performed at 200 RCF, 0 ˚C, for 10 min on a Thermo Fisher Scientific Sorvall ST 16R centrifuge unless otherwise noted or an Eppendorf (Hamburg, Germany) 5424R microcentrifuge. Isothermal calorimetry titrations were performed on an ITC200 instrument Malvern Panalytical (Malvern, United Kingdom). ATR-IR spectra (4000-650 cm^{-1}) were obtained using a Thermo Fisher Scientific Nicolet iS10 infrared spectrophotometer. Mass spectra were collected with a Sciex (Framingham, MA) 4800 MALDI TOF/TOF Analyzer in positive reflector mode. Spectra were analyzed using matrix peaks for alpha cyano-4-hydroxy-cinnamic acid (CHCA) or 2,5- Dihydroxybenzoic acid (DHBA), and the peptide, (Glu)-Fibrinopeptide human, as reference peaks for internal calibration. Concentration of sample was adjusted to give comparable intensity of the sample peaks to the reference peaks.

Synthetic Procedures:

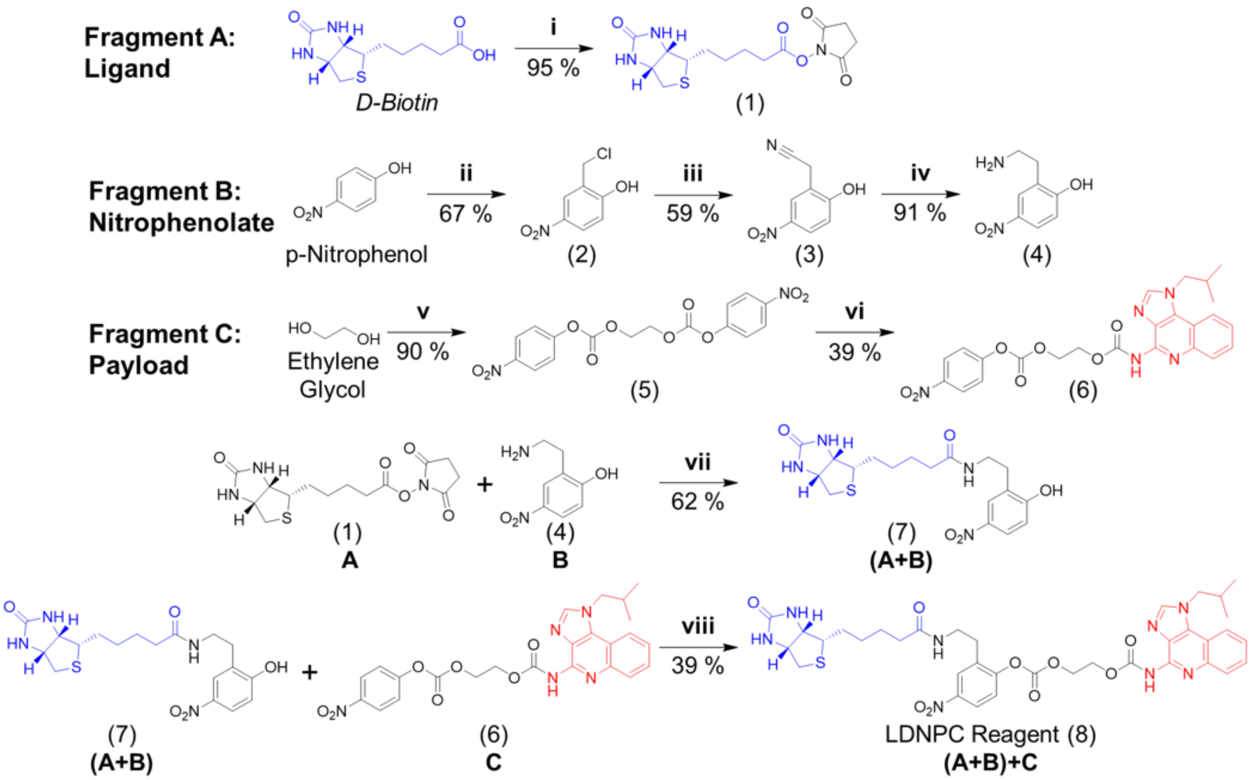

Scheme S1: Synthetic route to ligand-directed nitrophenol carbonate (LDNPC) reagent (8). **Fragment A i**: EDC-HCl, NHS, DMF, RT 24 h. **Fragment B ii**: Methylal, HCl (g), HCl (*aq*) conc., H_2SO_4 (*aq*) conc., 72 °C, 4 h. **iii**: acetonitrile, 5 M KCN in H_2O, 0 °C 30 min, 60 °C 30 min. **iv**: 1) BH_3-THF 1 M in THF, 100 °C, 4 h. 2) HCl 0.8 M in methanol, 100 °C, 12 h. **Fragment C v**: *p*-nitrophenyl chloroformate, pyridine, DCM, 90 °C, 24 h . **vi**: Imiquimod, THF, MW Irradiation: 90 °C, 50 min, 1 bar. **(A+B) vii**: DIPEA, DMF, RT, 18 h. **(A+B) + C viii**: DIPEA, RT, 24 h.

(1) *N*-Succinimidyl *D*-biotinate

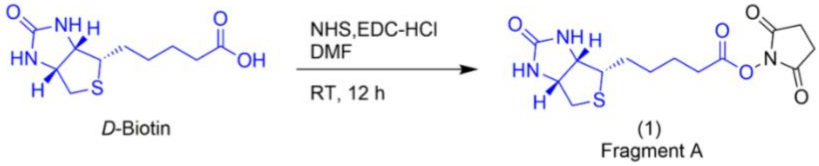

To a flame dried 100 mL round bottomed flask with stir bar was added *D*-Biotin (1.07 g, 4.38 mmol) and EDC·HCl (0.97 g, 5.06 mmol). The solids were dissolved in DMF (25 mL). To the stirred suspension was added NHS (0.53 g, 4.60 mmol) and left at RT. After 12 h, the contents of the flask are completely solubilized and the DMF is removed in vacuo (60 °C, 200 mtorr) to dryness. The white solid was washed with methanol (4 x 8 mL) and diethyl ether (50 mL) and dried under vacuum to yield N-Succinimidyl D-biotinate (**1**), a white amorphous solid (1.42 g, 95% yield). R_f=0.74, 25% methanol in DCM. ^{1}H NMR (400 MHz, [d_6]DMSO, 25 °C): δ=6.44 (s, 1H; NH), 6.38 (s, 1H; NH), 4.31 (dd, *J*=7.7, 5.0, 1H; CH), 4.15 (ddd, *J*=7.7, 4.4, 1.7, 1H; CH), 3.10 (ddd, *J*=8.1, 6.4, 4.3, 1H; CH), 2.88 – 2.74 (m, 5H; CH_2,CH_2, diastereotopic CH_2), 2.67 (t, *J*=7.4, 2H; CH_2), 2.58 (d, *J*=12.4, 1H; diastereotopic CH_2), 1.74 – 1.55 (m, 3H; CH_2, diastereotopic CH_2), 1.56 – 1.35 (m, 3H; CH_2, diastereotopic CH_2) ppm; ^{13}C NMR (101 MHz, [d_6]DMSO, 25 °C): δ=170.3, 169.0, 162.7, 61.0, 59.2, 55.3, 40.0, 30.0, 27.8, 27.6, 25.4, 24.3 ppm; UV/VIS (DMSO): λ_{Max}(ε)=258 nm (121) ; IR(ATR): ν=3232 (s), 2943 (m), 1820 (s), 1790 (s), 1748 (vs), 1730 (vs), 1704 (vs) cm^{-1}; HRMS (MALDI): m/z calcd for $C_{14}H_{19}N_3O_5S+H^+$: 342.1124 $[M+H]^+$, Observed 342.1130, (Δ=1.8 ppm).

(2) 2-(chloromethyl)-4-nitrophenol

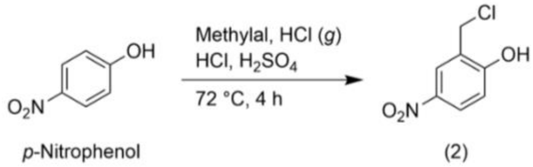

Compound (**2**) was prepared following the procedure outlined in Organic Synthesis.[1] The reaction was carried out on 50.0 g (0.359 mol) scale of p-nitrophenol. The outlined recrystallization procedure was foregone due to the collected precipitate having suitable purity by NMR following drying in vacuo. Isolated 2-(chloromethyl)-4-nitrophenol (**2**), a pale-yellow amorphous powder (45.3 g, 67% yield). R_f=0.32, 1% glacial acetic acid in DCM. ^{1}H NMR (600 MHz, [d_6]DMSO, 25 °C): δ=11.57 (s, 1H; OH), 8.27 (d, *J*=2.8, 1H; Ar-H), 8.08 (dd, *J*=9.0, 2.9, 1H; Ar-H), 7.02 (d, *J*=9.0, 1H; Ar-H), 4.74 (s, 2H; CH_2) ppm; ^{13}C NMR (151 MHz, [d_6]DMSO, 25 °C): δ=162.0, 139.3, 126.7, 126.2, 124.9, 115.8, 40.7 ppm; UV/VIS (Acetonitrile): λ_{Max}(ε)=204 nm (10100); IR(ATR): ν=3318 (s) cm^{-1}; HRMS (MALDI): m/z calcd for $C_7H_6ClNO_3-H^-$: 185.9958 $[M-H]^-$, Observed 185.9960, (Δ=1.1 ppm).

(3) 2-(2-hydroxy-5-nitrophenyl)acetonitrile

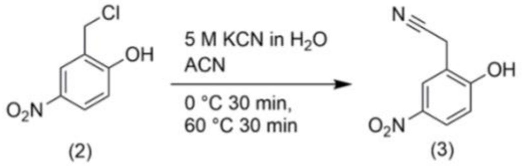

To a 500 mL round bottomed flask with stir bar was added a solution of KCN in water (45 mL, 5 M). An addition funnel was charged with (**2**) (1.53 g, 8.16 mmol) in acetonitrile (45 mL, 0.18 M). The solution was brought to 0 ˚C, and the organic solution was added dropwise over 80 min. After complete addition, the ice bath was removed, and solution heated to 60 ˚C. After 35 min the solution was removed from heat and again cooled to 0 ˚C in an ice bath before quenching with HCl (180 mL, 2 M). DCM (100 mL) was then added to the stirred solution before filtering off insoluble precipitate (ppt). The ppt was then washed with DCM (100 mL) and the biphasic filtrate transferred to a separatory funnel. The organic layer was separated before extracting the aqueous layer with DCM (100 mL). Combined organics were dried over Na_2SO_4 and solvent removed by rotary evaporation followed by vacuum (200 mTorr, 80 ˚C) to dryness. The crude material was purified with flash chromatography, Gradient Method: A: 1% glacial acetic acid in DCM, B: methanol, 0% B 1 column volumes (CV), 0 to 4% B over 12 CV. Chromatography solvent was removed in vacuo and the purified product was suspended in benzene and lyophilized to yield 2-(2-hydroxy-5-nitrophenyl)acetonitrile (**3**) as a pale-yellow amorphous powder (0.86 g, 59% yield). R_f=0.76, 5% methanol 1% glacial acetic acid in DCM. ^{1}H NMR (600 MHz, [d$_6$]DMSO, 25 °C): δ=11.67 (s, 1H; OH), 8.24 (d, *J*=2.8, 1H; Ar-H), 8.12 (dd, *J*=9.0, 2.9, 1H; Ar-H), 7.03 (d, *J*=9.0, 1H; Ar-H), 3.93 (s, 2H; CH_2) ppm; ^{13}C NMR (151 MHz, [d$_6$]DMSO, 25 °C): δ=161.6, 139.4, 125.7, 125.7, 119.0, 118.2, 115.3, 17.9 ppm; UV/VIS (Acetonitrile): λ_{Max}(ε)=204 nm (11800); IR(ATR): ν=3219 (s), 2271 (m) cm^{-1}; HRMS(MALDI): m/z calcd for $C_8H_6N_2O_3+H^+$: 179.0457 [M+H]$^+$, Observed 179.0464, (Δ=3.9 ppm).

(4) 2-(2-aminoethyl)-4-nitrophenol

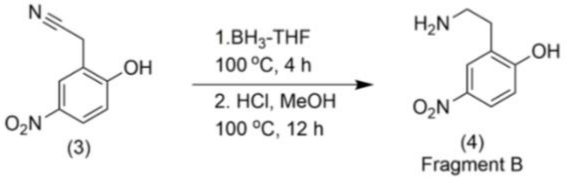

To a flame dried 250 mL round bottomed flask with stir bar and jacketed condenser was added (**3**) (1.02 g, 5.73 mmol). The solid was dissolved in 1 M BH_3·THF (31 mL, 31 mmol) and brought to reflux at 100 ˚C. After 4 h, the solution was removed from heat and a solution of HCl in methanol (72 mL, 0.8 M) was added with caution of exotherm and the solution was again brought to reflux at 100˚C. After 12 h the solution was cooled and concentrated by rotary evaporation to a syrup. This crude syrup was purified by flash chromatography. Gradient method: A: DCM, B: methanol, 10% B 3 CV, 10 to 25% B 12 CV, 25% B 3 CV. The chromatography solvent was removed in vacuo to yield 2-(2-aminoethyl)-4-nitrophenol (**4**) as a yellow crystalline solid (0.95 g,

91% yield). R_f=0.23, 20% methanol in DCM. ^{1}H NMR (600 MHz, [d_6]DMSO, 25 °C): δ=11.62 (s, 1H; OH), 8.16 (s, 2H; NH_2), 8.05 (d, *J*=2.9, 1H; Ar-H), 8.03 (dd, *J*=8.8, 2.9, 1H; Ar-H), 7.15 (d, *J*=8.8, 1H; Ar-H), 3.02 (t, *J*=7.6, 2H; CH_2), 2.95 – 2.89 (m, 2H; CH_2) ppm; ^{13}C NMR (151 MHz, [d_6]DMSO, 25 °C): δ=162.4, 139.3, 126.4, 124.7, 124.6, 115.3, 37.8, 27.8 ppm; UV/VIS (Methanol): $λ_{Max}$(ε)=202 nm (95600); IR(ATR): ν=3124 (s), 3053 (s), 2950 (s) cm^{-1}; HRMS(MALDI): m/z calcd for $C_8H_{10}N_2O_3+H^+$: 183.0770 $[M+H]^+$, Observed 183.0771, (Δ=0.5 ppm)

(5) ethane-1,2-diyl bis(4-nitrophenyl carbonate)

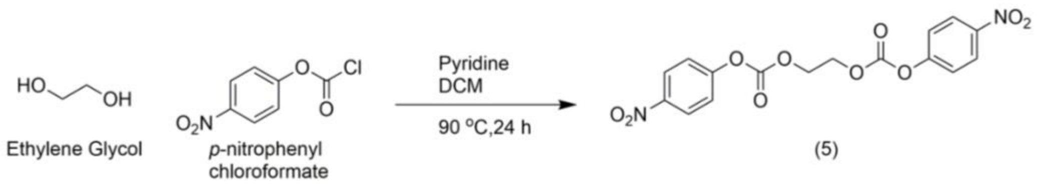

To a flame dried 100 mL round bottomed flask with stir bar and jacketed condenser was added *p*-nitrophenyl chloroformate (7.14 g, 35.4 mmol) and dissolved in DCM (40 mL). To the solution was added ethylene glycol (901 µL, 1.00 g, 16.1 mmol) followed by pyridine (3.00 mL, 37.1 mmol) and the solution brought to reflux at 90 ˚C. After 24 h the reaction is cooled to RT and poured into water (200 mL). The biphasic solution is extracted with DCM (3 x 50 mL) and the combined organics washed with brine (200 mL) prior to drying over Na_2SO_4. Solvent is removed in vacuo and the crude oil purified by flash chromatography and loaded onto the column in DCM. Gradient method: A: hexanes, B: ethyl acetate, 20% B 2.5 CV, 20% to 50% B over 14 CV, 50% to 100% B over 0 CV, 100% B 6 CV. The chromatography solvent was removed in vacuo and the product lyophilized from benzene to yield (**5**) as a white amorphous powder (5.67 g, 90% yield). R_f=0.74, 50% ethyl acetate in hexanes. ^{1}H NMR (400 MHz, [d_1]$CHCl_3$, 25 °C): δ=8.24 (d, *J*=9.2, 1H; Ar-H), 7.36 (d, *J*=9.1, 1H; Ar-H), 4.59 (s, 1H; CH_2) ppm; ^{13}C NMR (101 MHz, [d_1]$CHCl_3$, 25 °C): δ=155.3, 152.3, 145.5, 125.3, 121.8, 66.2 ppm; UV/VIS (Acetonitrile): $λ_{Max}$(ε)=208 nm (47000); IR(ATR): ν=1754 (vs) cm^{-1}; HRMS(MALDI): m/z calcd for for $C_{16}H_{12}N_2O_{10}+Na^+$: 415.0390 $[M+Na]^+$, Observed 415.0401, (Δ=2.6 ppm).

(6) 2-(4-nitrophenyl carbonate)ethyl (1-isobutyl-1H-imidazo[4,5-c]quinolin-4-yl)carbamate

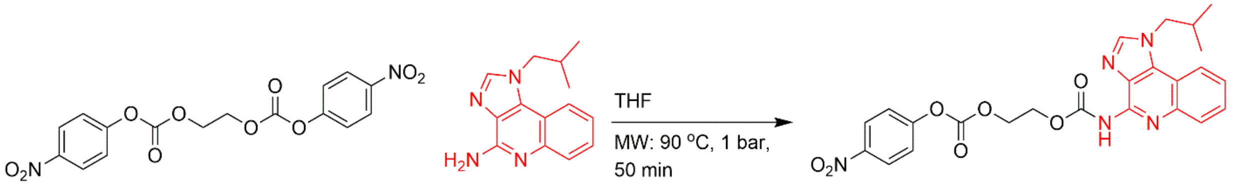

(5) Imiquimod (6) Fragment C

To a flame dried 5 mL microwave reaction vial with stir vane was added Imiquimod (200 mg, 0.83 mmol) and compound (**5**) (410 mg, 1.04 mmol). The solids were suspended in THF (5.5 mL). The vial was sealed with a septum and transferred to a microwave reactor. The solution was irradiated for 50 mins (Power Max:120 W, Power Average: 22 W, Temp: 90 ˚C, Average Pressure: 1 bar, Pre-stirring: 30 sec, Cooling: off) and then cooled to RT. The solution was concentrated to a crude oil, and purified by flash chromatography, loaded onto the column in DCM. Gradient method: A: hexanes, B: ethyl acetate, 60% B 2 CV, 60% to 100% B over 20 CV,100% B 6 CV. Chromatography solvent was removed in vacuo and the product lyophilized from benzene to yield (**6**) as a white amorphous powder (161 mg, 39% yield). R_f=0.26, 80% ethyl acetate in hexanes. ^{1}H NMR (600 MHz, [d_6]DMSO, 25 °C): δ=9.98 (s, 1H; NH), 8.35 (s, 1H; Ar-H), 8.27 (d, *J*=9.1, 2H; Ar-H), 8.23 (d, *J*=8.2, 1H; Ar-H), 7.98 (d, *J*=8.3, 1H; Ar-H), 7.66 (t, *J*=7.6, 1H; Ar-H), 7.61 (t, *J*=7.6, 1H; Ar-H), 7.57 (d, *J*=9.0, 2H; Ar-H), 4.55 – 4.51 (m, 2H; CH_2), 4.49 – 4.45 (m, 4H; CH_2, CH_2), 2.18 (dh, *J*=13.1, 6.5, 1H; CH), 0.91 (d, *J*=6.6, 6H; CH_3) ppm; ^{13}C NMR (151 MHz, [d_6]DMSO, 25 °C): δ=155.2, 152.8, 152.0, 145.1, 144.7, 144.3, 143.0, 133.4, 131.3, 128.9, 127.3, 125.4, 125.3, 122.5, 120.8, 116.8, 67.4, 62.1, 53.5, 28.4, 19.3 ppm; UV/VIS (DMSO): λ_{Max}(ε)=258 nm (150000); IR(ATR): v=1774 (s), 1739 (s) cm^{-1}; HRMS(MALDI): m/z calcd for $C_{24}H_{23}N_5O_7+H^+$: 494.1676 $[M+H]^+$, Observed 494.1660, (Δ=3.2ppm)

(7) *N*-(2-hydroxy-5-nitrophenethyl)-*D*-biotinamide

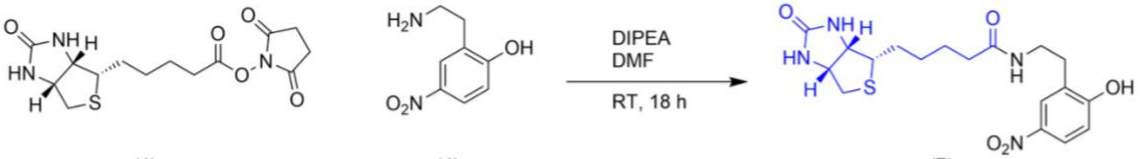

(1) Fragment A (4) Fragment B (7) A+B

To a flame dried 100 mL round bottomed flask with stir bar was added (**1**) (1.23 g, 3.60 mmol) and (**4**) 406 mg, 2.23 mmol) which were suspended in DMF (14 mL). DIPEA (1.162 mL, 6.67 mmol) was added and the solution stirred at RT. After 12 h, DMF was removed in vacuo and the crude material was concentrated onto 5 g of silica gel to solid load for flash chromatography. Gradient Method: A: DCM, B: methanol, 8% B for 1 CV, 8% to 10% B for 12 CV, 10% B for 5 CV. The chromatography solvent was removed by rotary evaporation to yield (**7**) as a yellow solid (564 mg, 62% yield). R_f=0.33, 5% methanol 1% glacial acetic acid in DCM. ^{1}H NMR (600 MHz, [d_6]DMSO, 25 °C): δ=11.45 (s, 1H; OH), 7.98 (dd, *J*=8.9, 2.9, 1H; Ar-H), 7.94 (d, *J*=2.9, 1H; Ar-H), 7.92 (t, *J*=5.6, 1H; NH), 7.12 (d, *J*=8.9, 1H; Ar-H), 6.42 (s, 2H; NH, NH), 4.30 (dd, *J*=7.7, 4.9, 1H; CH), 4.11 (dd, *J*=7.7, 4.4, 1H; CH), 3.27 (q, *J*=6.7, 2H; CH_2), 3.05 (ddd, *J*=8.5, 6.3, 4.4, 1H:CH), 2.81 (dd, *J*=12.4, 5.1, 1H; diastereotopic CH_2), 2.71 (t, *J*=7.0, 2H; CH_2), 2.58 (d, *J*=12.4, 1H;

diastereotopic CH_2), 2.01 (t, *J*=7.3, 2H; CH_2), 1.58 (ddt, *J*=12.6, 9.6, 6.2, 1H; diastereotopic CH_2), 1.44 (dddd, *J*=22.8, 14.2, 11.0, 6.2, 3H; CH_2, diastereotopic CH_2), 1.24 (ddt, *J*=15.1, 10.8, 4.6, 2H; CH_2) ppm; ^{13}C NMR (151 MHz, [d_6]DMSO, 25 °C): δ=172.5, 163.2, 162.9, 139.5, 127.3, 126.5, 124.3, 115.5, 61.4, 59.7, 55.8, 40.3, 38.1, 35.7, 30.0, 28.6, 28.4, 25.7 ppm; UV/VIS (DMSO): $\lambda_{Max}(\varepsilon)$=332 nm (7460); IR(ATR): ν=3350 (s), 3247 (m), 1716 (s) cm^{-1}; HRMS(MALDI): m/z calcd for $C_{18}H_{24}N_4O_5S+H^+$: 409.1546 $[M+H]^+$, Observed 409.1560, (Δ=3.4 ppm).

(8) 2-(4-nitro-2-(2-N-ethyl-D-biotinamide)phenyl carbonate)ethyl (1-isobutyl-1H-imidazo[4,5-c]quinolin-4-yl)carbamate

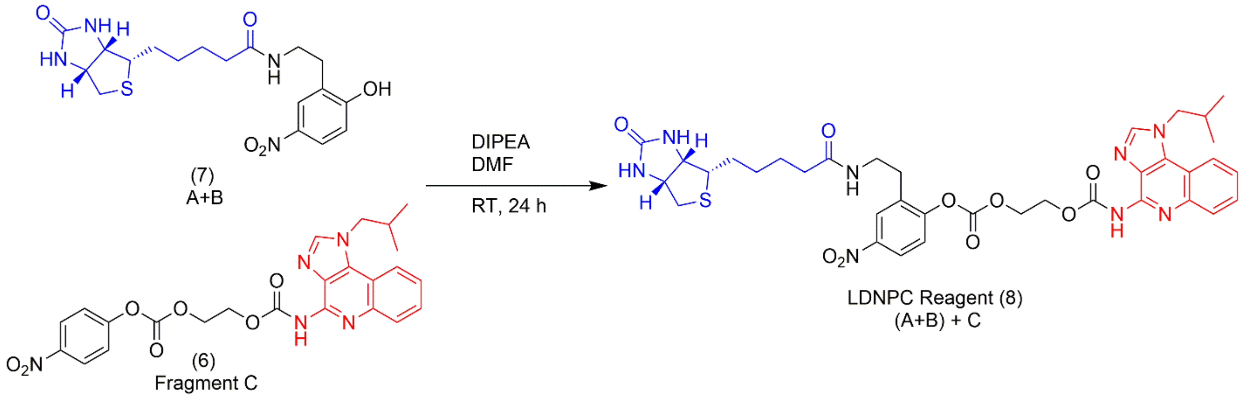

To an oven dried 5 mL conical flask was added (**6**) (81 mg, 0.16 mmol) and dissolved in DMF (2.2 mL). Next, DIPEA (42 µL, 0.24 mmol) was added followed by addition of (**7**) (101 mg, 0.25 mmol) and the solution was stirred at RT. After 24 h, equilibrium is reached and the DMF removed in vacuo (250 mTorr, 60 °C). The residue was dissolved in 30% acetonitrile in water and purified by reverse-phase flash chromatography. Gradient method: A: Water, B: Acetonitrile, 10% B 2 CV, 10% to 100% B over 20 CV, 100% B 5 CV. Fractions containing the product were immediately flash frozen and lyophilized to yield compound (**8**) as a white amorphous powder (48 mg, 39% yield). R_f=0.77, 10% methanol in DCM. ^1H NMR (600 MHz, [d_6]DMSO, 25 °C): δ=9.98 (s, 1H; NH), 8.36 (s, 1H; Ar-H), 8.25 (d, *J*=7.6, 1H; Ar-H), 8.17 (d, *J*=2.9, 1H; Ar-H), 8.12 (dd, *J*=8.9, 2.8, 1H; Ar-H), 7.98 (d, *J*=8.2, 1H; Ar-H), 7.85 (t, *J*=5.7, 1H; NH), 7.67 (t, *J*=7.2, 1H; Ar-H), 7.62 (t, *J*=7.6, 1H; Ar-H), 7.58 (d, *J*=8.9, 1H; Ar-H), 6.38 (s, 1H; NH), 6.34 (s, 1H; NH), 4.57 – 4.52 (m, 2H; CH_2), 4.51 – 4.44 (m, 4H; CH_2, CH_2), 4.28 (dd, *J*=7.7, 5.1, 1H; CH), 4.07 (ddd, *J*=7.3, 4.5, 1.8, 1H; CH), 3.29 (q, *J*=6.6, 2H; CH_2), 3.00 (ddd, *J*=8.7, 6.2, 4.4, 1H; CH), 2.84 – 2.73 (m, 3H; CH_2, diastereotopic CH_2), 2.56 (d, *J*=12.4, 1H; diastereotopic CH_2), 2.20 (dh, *J*=13.4, 6.7, 1H; CH), 1.96 (t, *J*=7.6, 2H; CH_2), 1.54 (ddt, *J*=12.7, 9.7, 6.3, 1H; diastereotopic CH_2), 1.40 (qt, *J*=14.4, 8.2, 3H; CH_2, diastereotopic CH_2), 1.26 – 1.10 (m, 2H; CH_2), 0.93 (d, J=6.6, 6H; CH_3) ppm; ^{13}C NMR (151 MHz, [d_6]DMSO, 25 °C): δ=172.1, 162.7, 153.7, 152.8, 152.0, 145.0, 144.7, 144.3, 142.9, 133.4, 133.3, 131.2, 128.9, 127.3, 125.9, 125.4, 123.5, 123.0, 120.8, 116.8, 67.5, 62.1, 61.0, 59.2, 55.3, 53.5, 39.6, 38.0, 35.2, 29.2, 28.4, 28.1, 27.9, 25.2, 19.3 ppm; UV/VIS (Acetonitrile): $\lambda_{Max}(\varepsilon)$=206 nm (9720); IR(ATR): ν=3281 (m), 2934 (m), 1762 (m), 1699 (s) cm^{-1}; HRMS(MALDI): m/z calcd for $C_{36}H_{42}N_8O_9S+H^+$: 763.2874 $[M+H]^+$, Observed 763.2896, (Δ=2.9 ppm).

Titration Protocol:

For titration of (**7**) and *p*-nitrophenol, a solution of NaOH (theoretical 5 mM) was first prepared by adding 50% (w/w) aqueous NaOH (264 µL) to water and diluting to a final volume of 1 L. The solution was standardized against potassium hydrogen phthalate (10 – 20 mg) dissolved in water (20.0 mL) with phenolphthalein (200 µL, 1 mg/mL H_2O:EtOH) as an endpoint indicator in quintuplet. After standardization, the sodium hydroxide solution was determined to be 3.09 ±0.04 mM. This solution was stored in a tightly capped bottle sealed with parafilm and used as titrant for all titrations over the course of 3 days. For titrations of (**7**) and *p*-nitrophenol, solutions in DMSO (61.8 mM) were prepared and for each run 1.50 mL of the respective stock solution was added to 100 mL of water for a working concentration of 0.913 mM. 50 mL of titrant was used for each run and burette reading and pH readings were recorded to the hundredths place. Drift of the pH meter was monitored by recording value of pH 7 buffer before and after each titration, in all cases drift was < 0.10 pH. Data was plotted and both the eye method and Gran plot were used to determine the endpoint. For (**7**) and PNP, both methods gave agreeable values to within 0.1 pH unit. Experimental pK_a for (**7**)=8.02 and for PNP=7.25 (+0.10 from theoretical value [2] **Figure S1**).

Hydrolysis Assay Protocols:

Buffer Preparation:

For all hydrolysis assays, 250 mL of a 50 mM citrate phosphate buffer was prepared from varied ratios of citric acid monohydrate and disodium phosphate to achieve pH 4,5,6 and 7. Before use, an aliquot of buffer was transferred to a centrifuge tube and adjusted with 1 M HCL or 1 M NaOH to the indicated pH ± 0.05. The standardized buffers were used for hydrolysis experiments with and without avidin.

Assay Without Avidin:

Samples were prepared at pH 4,5,6 & 7 by first loading the buffer (3.900 mL) into a HPLC sample vial sealed with a septum and equilibrating the buffer to 37.0 ˚C in the HPLC autosampler tray. At t_0 a 10 mM solution of LDNPC (**8**) in DMSO (100 µL) was added to the warm buffer to give a 250 µM working solution. At the indicated timepoint, the autosampler loaded 25 µL of sample and ran the following gradient method: 20% B 10 min, 20% to 95% B 6 min, 95% B for 3 min. The resulting chromatographic peaks for Imiquimod were integrated and the peak area converted to concentration via a 6-point calibration curve ran in triplicate (0 – 250 µM). To simplify the graph, these concentrations were expressed as % of Imiquimod released, based on the theoretical max of 250 µM (**Figure S2)**.

Carbonate Cleavage Kinetics:

Bond cleavage of (**8**) to (**7**) was measured at 405 nm in the presence of avidin, biotin and avidin, buffer, and bovine serum albumin (BSA). 50 µL of (**8**) (1200 µM in DMSO) was added to a cuvette containing 450 µL of either: avidin (0.42 mg/mL), avidin + biotin (0.42 mg/mL, 12 µM, mixed 10 min prior to kinetic run), BSA (0.42 mg/mL) or citrate phosphate buffer (50 mM, pH 7.0). All protein solutions were made in citrate phosphate buffer (50 mM, pH 7.0). Kinetic assay run parameters were set to measure absorbance at 405 nm, every 1 min, for 90 min. Assays were run in triplicate at 22 ˚C. Absorbance values were converted to concentration via a 5-point calibration curve ran in triplicate (0 – 100 µM). Concentrations were subtracted from the theoretical maximum of (**8**) (120 µM) to arrive at the degradation curve shown. Significance was evaluated by two-tailed student T-test (**Figure S3).**

Assay with Avidin:

Samples were prepared for pH 7 with avidin, and without avidin as control. Avidin (25 mg) was dissolved in 1 mL of pH 7 buffer to yield a 25 mg/mL solution. To a HPLC sample vial sealed with a septum were loaded with 1.5 mL of buffer (w/ and w/o avidin) and equilibrated to 37.0 ˚C in the HPLC autosampler tray. At t_0 a 10 mM solution of LDNPC (**8**) in DMSO (38.5 µL) was added to the warm buffer to give a 250 µM working solution. At each timepoint, the vial was removed from the HPLC autosampler, 115 µL loaded into a 10kDa MWCO CFD , and spun at 14,000 RCF, 37 ˚C, 5 min. Following centrifugal separation of biomolecule, 95 µL of filtrate was loaded into the HPLC autosampler and the autosampler loaded 25 µL of sample and ran the following gradient method: 20% B 10 min, 20% to 95% B 6 min, 95% B for 3 min. The resulting chromatographic peaks for Imiquimod were integrated and the peak area converted to concentration (**Figure S4)** via a 6-point calibration curve following the same centrifugal filtration protocol (0 – 250 µM).

Avidin Crystallization and Structure Determination Protocol:

The crystals of Avidin-(**7**) were grown by hanging drop method at 300 K. The pure protein (5 mg mL^{-1} in 100 mM Tris, pH 7.5) was mixed with an equal volume of the reservoir solution (0.1 M Tris, pH 7.0, and 25% (w/v) PEG 1,500). The rod-shaped and yellow-colored crystals started to appear after 2 weeks. Avidin-(**7**) crystallized in $P2_1$ space group, which diffracted up to 1.58 Å resolution. Diffraction data were collected at the Advanced Light Source (ALS) beamline 5.0.2 with a wavelength of 1.0 Å. The software package HKL2000[3] was used for processing diffraction data. Description of the diffraction data is listed in Table S1. Initial phasing for diffraction data was performed by molecular replacement with the PHENIX Phaser[4] using the crystallographic coordinates of avidin from *Gallus gallus* complexed with biotin (PDBID: 2AVI) as a search model. Refinement and model building were done using PHENIX and Coot[5]. The final crystallographic R_{work} and R_{free} were 20.49% and 24.14%, respectively. The root mean square deviations from ideal geometry of the model were 0.013 Å for bonds and 1.75° for angles. The refinement statistics are listed in Table S1. Crystallographic data and coordinates were deposited in the Protein Data Bank (PDBID: 6XND).

Isothermal Titration Calorimetry Protocol:

Isothermal calorimetry titrations were performed for Avidin and compounds (**7**) and (**8**). The concentration of protein in the calorimetric titration cell was diluted to 75 µM in the appropriate buffer. All titrations were performed at 25 ˚C with a stirring speed of 750 rpm and 27 injections (1.4 µL per injection). Compound (**7**) was brought to a concentration of 0.75 mM in 100 mM Tris buffer, pH 8.0, injected into the protein solution, and the heat of binding recorded. (**Figure S5)** For comparisons of compound (**7**) and (**8**), titrations were conducted at a concentration of 1.50 mM in 40% DMSO (required to solubilized (**8**)) in 100 mM Tris buffer pH 7.0, and injected into the protein solution, and the heat of binding recorded. (**Figure S6**).

HABA Competition Assay Protocol:

A competitive binding assay using 4'-hydroxyazobenzene-2-carboxylic acid (HABA) was done. The assay was conducted using 2.0 µM Avidin, 15 µM HABA in 0.1 M sodium phosphate buffer pH 6.5. This solution was titrated using 300 µM solutions of either (**7**) or Biotin in 10 mM NaOH. For (**8**), the solution was 25% DMSO and 10 mM NaOH. Changes in absorbance were measured

at 500 nm on a plate reader. All assays were done in triplicate. K_d of compounds was measured using the Cheng-Prusoff equation[6] (**Figure S7**).

Cell Culture Protocols:

RAW-Blue Macrophages: As per manufacturer instructions, RAW-Blue cells (modified RAW264.7 macrophages, Invivogen, CA) were grown in complete culture media composed of DMEM with 4.5 g L^{-1} glucose, 2 mM L-glutamine, 100 U mL^{-1} PenStrep, and 0.1 mg mL^{-1} Zeocin supplemented with 10% HI-FBS. Media was changed every 3-4 days, and cells were passaged once per week. Passaging involved changing media, counting, and seeding 3 x 10^5 cells in 35 mL of new complete media in a new T-175 culture flask.

RAW-Blue Assay Protocol:

Measurement of RAW-Blue cell activation was performed similar to manufacturer instructions (Invivogen, CA). To measure NF-κB transcription via RAW-Blue assay, RAW-Blue cells were seeded in an optically transparent bottomed 96-well plate in 180 µL of complete cell media at a density of 1 x 10^5 cells $well^{-1}$. Prodrug (**8**) and Imiquimod were diluted in DPBS and administered to the wells at varying doses (1 - 100 µM) and total well volumes were adjusted to 200 µL with complete cell media. Plates were incubated for 16 h before measuring NF-κB transcription by colorimetric assay of secreted alkaline phosphatase (See Secreted Alkaline Phosphatase Colorimetric Assay Protocol) (**Figure S8**).

Secreted Alkaline Phosphatase Colorimetric Assay Protocol:

Detection Media: Quanti-Blue detection media was used to measure Secreted Alkaline Phosphatase for all experiments.

Quanti-Blue: 1.168 mg/mL BCIP (5-bromo-4-chloro-3'-indolyphosphate) *p*-toluidine salt (VWR) in 1M aqueous diethanolamine.

To an optically transparent bottomed 96-well plate was added 180 µL of detection media per well. Next, 20 µL of supernatant from RAW-Blue immune reporter cells was added to each well and the plate was incubated for 1-6 h. After this time, the absorbance was measured (620 nm Quanti-Blue) on a microplate reader. Experiments were run in triplicate and a blank was subtracted from all values acquired.

Time Dependent Immunogenicity Assay Protocol:

This assay was designed around saturating avidin with LDNPC (**8**). To this end, based on the activity definition that avidin binds 7 µg Biotin/ mg Avidin, at each timepoint in a 10kDa MWCO CFD , a 17.45 mg/mL solution of avidin in PBS (200 µL) was loaded with a 10 mM solution of LDNPC (**8**) dissolved in DMSO (13 µL). The solution was incubated for 60 s at 37 ˚C before the protein and filtrate were separated by centrifuging at 37 ˚C, 14,000 RCF, 5 min. After the initial separation, the retained avidin-LDNPC (**8**) complex, was washed three times by repeating the following procedure: PBS (300 µL) was added, the solution vortexed and the protein and filtrate separated by centrifuging at 37 ˚C, 14,000 RCF, 5 min. After the final wash cycle the avidin-LDNPC (**8**) complex was reconstituted in PBS (200 µL), vortexed and transferred by pipette to a 96 well plate and incubated (37 °C, 5% CO_2) for the indicated time (72-0 h). After incubation, 20

μL of this solution was added to 180 μL of RB cell suspension and incubated for 16 h at a theoretical 50 μM concentration after 1:10 dilution into the cell media. NF-κB transcription was then measured following the RAW-Blue Assay Protocol outlined above. Blanks and controls were treated with the same procedure outlined for LDNPC (**8**) above except for positive Imiquimod controls (10 – 100 μM) which were added directly to the RAW-Blue assay (**Figure 4b**).

Supporting Figures and Tables:

Table S1. X-ray diffraction data and refinement statistics for Avidin-(7)

**Data collection**	
Space group	P1 2_1 1
Cell dimensions	
a, b, c (Å)	46.526,79.254, 74.683
α, β, γ (°)	90.00, 105.456, 90.00
Resolution (Å)	43.64- 1.589 (1.646- 1.589)
R_{merge}	0.08059 (1.367)
Wavelength (Å)	1
Unique reflections	70248 (6991)
Completeness (%)	99.90 (99.63)
$<I>/\sigma I$	10.23 (1.09)
CC1/2	0.999 (0.462)
Redundancy	6.4 (6.1)
**Refinement**	
R_{work} / R_{free}	0.2049/ 0.2414 (0.3342/ 0.3644)
Number of atoms	4257
Protein and ligand	3952
Water	305
B-factors (Å^2)	
All atoms	30.99
Solvent	40.39
R.m.s deviations	
Bonds (Å)	0.013
Angles (º)	1.75
Ramachandrans	
% Favored	98.11
% Outliers	0.63
Clash score	11.84

Statistics for the highest-resolution shell are shown in parentheses.

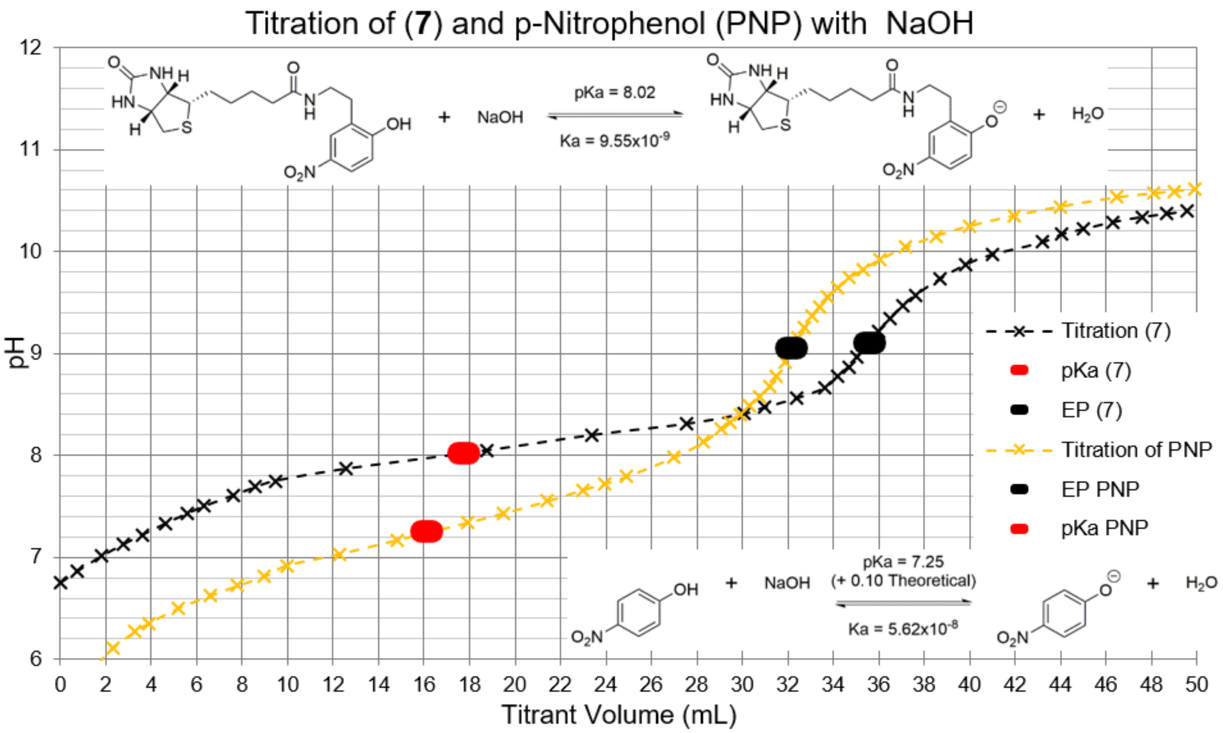

Figure S1: Titration of nitrophenol (**7**) (yellow) and *p*-nitrophenol (PNP, black) with 3.09 mM NaOH. The titration endpoints for the corresponding traces are shown (black ovals) and the calculated half equivalence point (pK_a) are shown (red ovals).

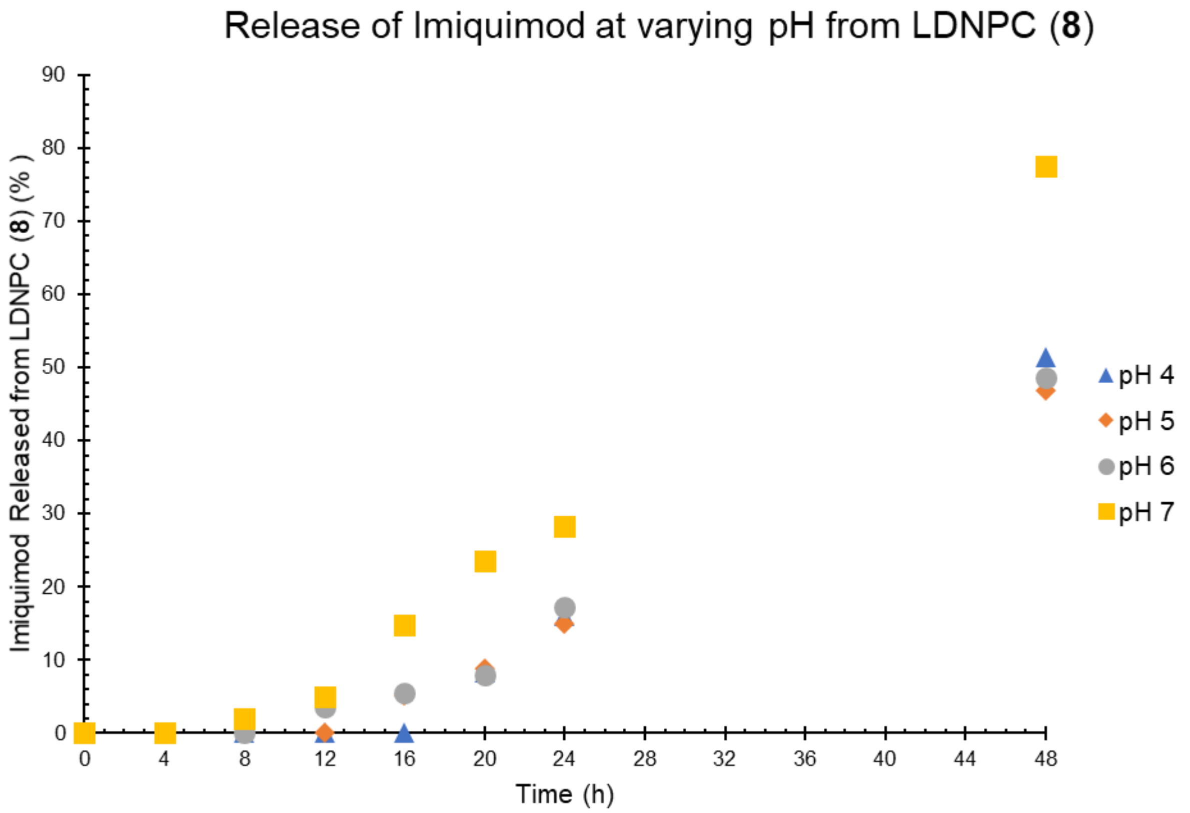

Figure S2: Release of Imiquimod from LDNPC (**8**) at pH 4 (blue triangles), 5 (orange diamonds), 6 (grey circles), and 7 (yellow squares). Imiquimod released was below the limit of detection until the 8 h timepoint.

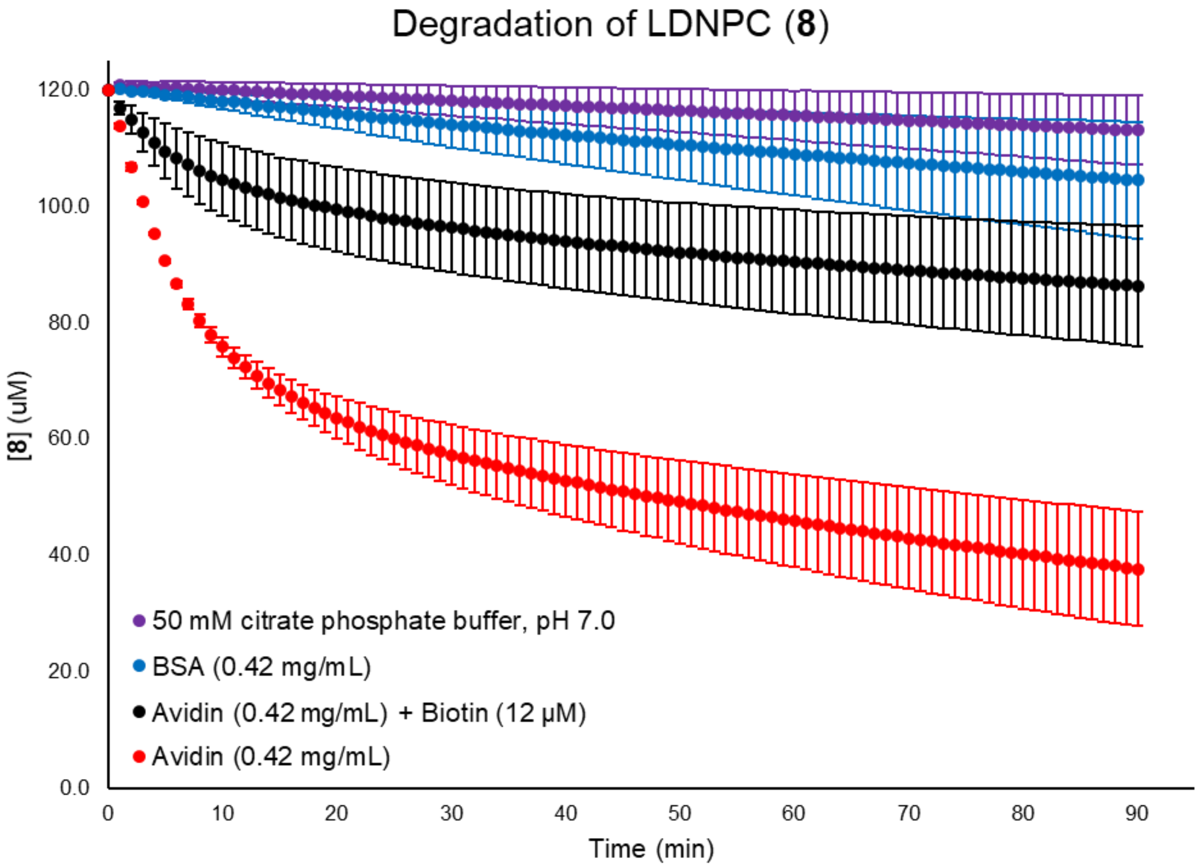

Figure S3: The kinetics of the second step of LDNPC chemistry was measured by monitoring the release of nitrophenolate (**7**) from LDNPC (**8**) (120 μM) in the presence of Avidin (Red, 0.42 mg/mL), Avidin + Biotin (Black, 0.42 mg/mL, 12 uM), Bovine Serum Albumin (BSA) (Cyan, 0.42 mg/mL) or citrate phosphate buffer (Purple, 50 mM pH 7). The effects of avidin on LDNPC (**8**) are statistically significant compared to BSA and Buffer $p < 0.05$ calculated at 2, 5, 10, 15, 20, 30, 60, and 90 min.

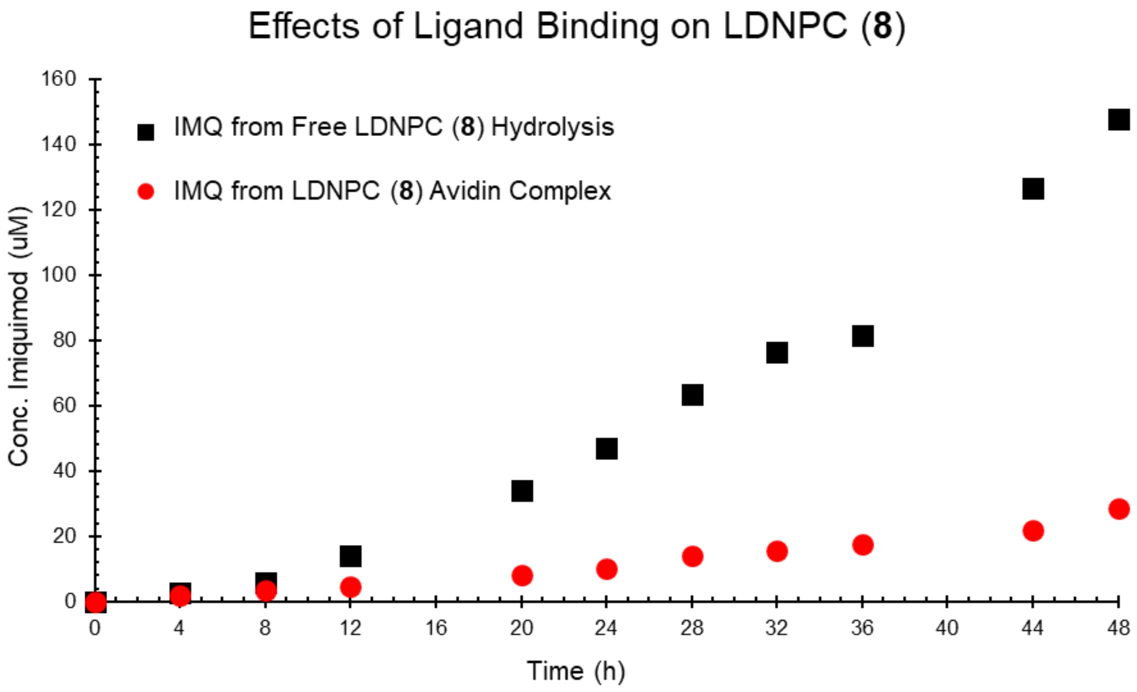

Figure S4: Release of Imiquimod from LDNPC (**8**) at pH 7 without avidin (black squares) and with avidin (red circles). It is hypothesized that the slower release of Imiquimod when avidin is added is attributed to the successful covalent labeling via LDNPC (**8**). The resulting carbonate or carbamate from labeling would be more stable than the carbonic acid resulting from direct hydrolysis of LDNPC (**8**).

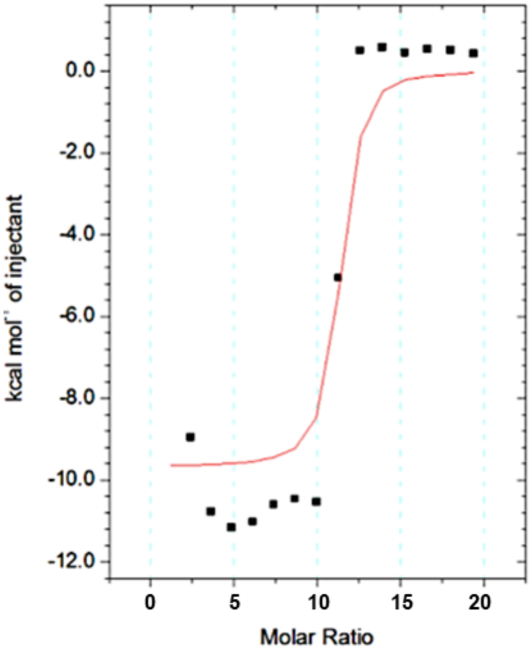

Figure S5: Determination of K_d using Isothermal Calorimetry. The K_d of (**7**) for avidin in pH 8.0 Tris buffer was calculated to be 2.25×10^{-7} M, $\Delta H = -9672$ cal mol^{-1}, $\Delta S = 2.02$ cal mol^{-1} k^{-1}.

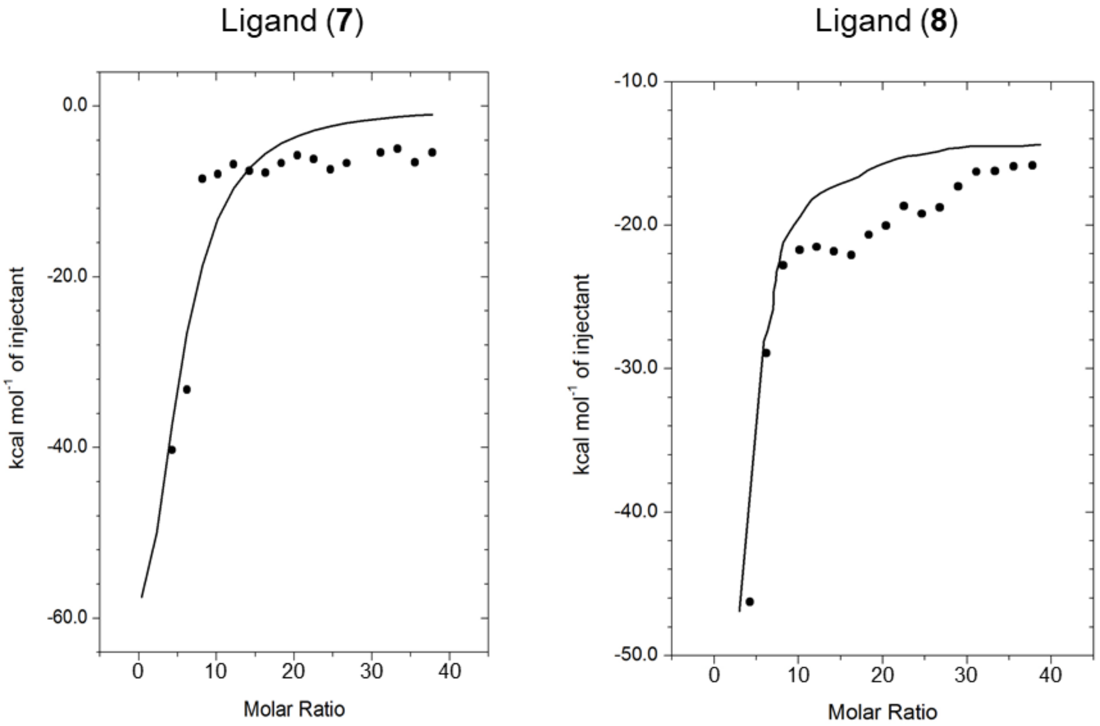

Avidin / Ligand	K_d (M)	ΔH (cal mol^{-1})	ΔS (cal mol^{-1} k^{-1})
Avidin / (**7**)	1.06×10^{-5}	-9.49×10^{-4}	-295
Avidin / (**8**)	9.52×10^{-5}	-7.17×10^{-4}	-222

Figure S6: Comparison of the affinities of (**7**) and (**8**) to avidin using Isothermal Calorimetry in pH 7.0 Tris buffer with 40% DMSO required to solubilized (**8**) at the required concentration. The K_d of both ligands agree to the same order of magnitude (10^{-5} M).

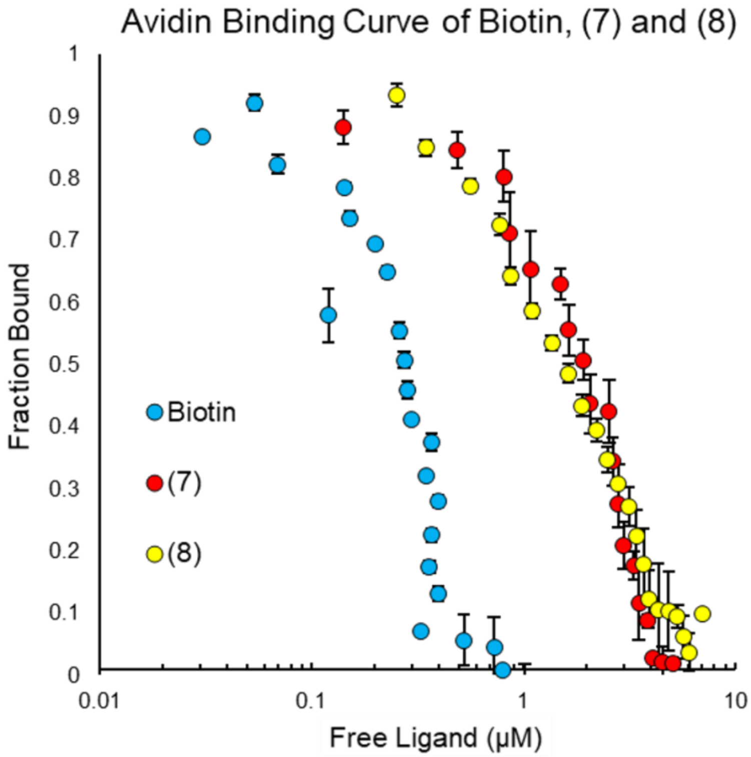

Figure S7: Ligand binding data using HABA assay. K_d of each ligand calculated as: Biotin: $1.90 \times 10^{-8} \pm 0.008$ M. (**7**): $1.36 \times 10^{-7} \pm 0.018$ M. (**8**): $1.04 \times 10^{-7} \pm 0.013$ M. Significant difference in binding between all comparisons of ligands ($p<0.05$).

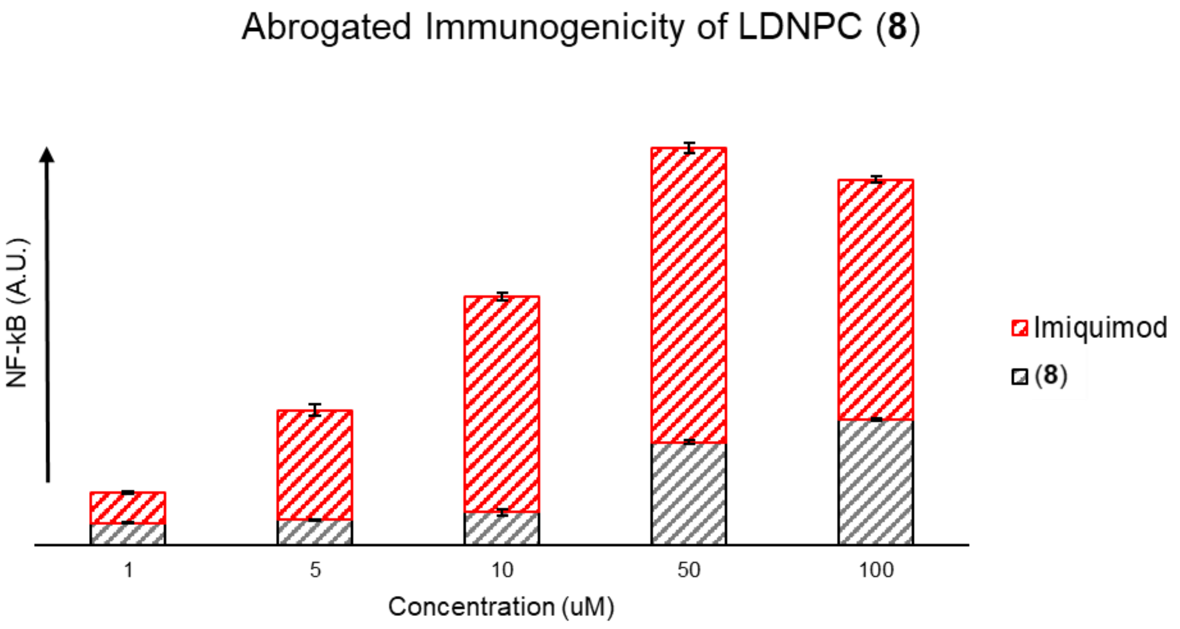

Figure S8: Comparison of the effect of equimolar concentrations of Imiquimod (red bars) and LDNPC (**8**) (grey bars) on Raw-Blue murine macrophages. It can be concluded that LDNPC (**8**) exhibits abrogated immunogenicity compared to the parent drug Imiquimod across all concentrations tested. It should be noted that the assay takes 16 h of incubation and that based on **Figure S2** some nonspecific hydrolysis of (**8**) to imiquimod was expected.

Characterization Data:

^{1}H NMR spectra for compound (**1**)

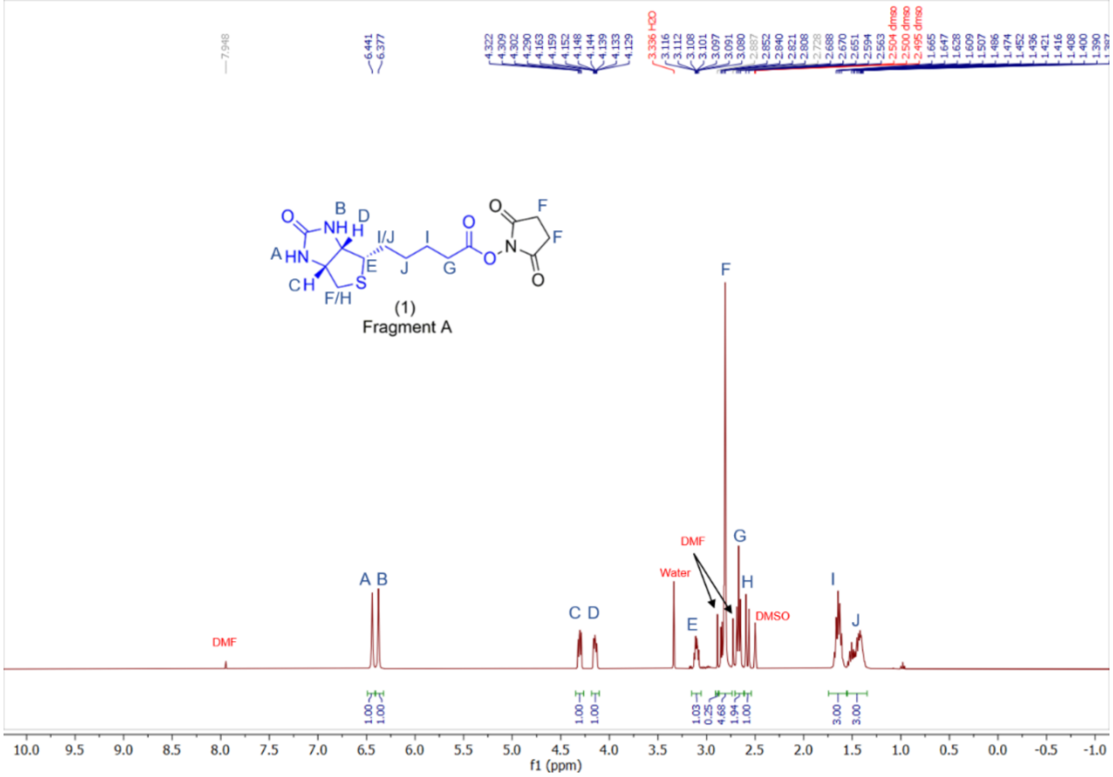

^{13}C NMR spectra for compound (**1**)

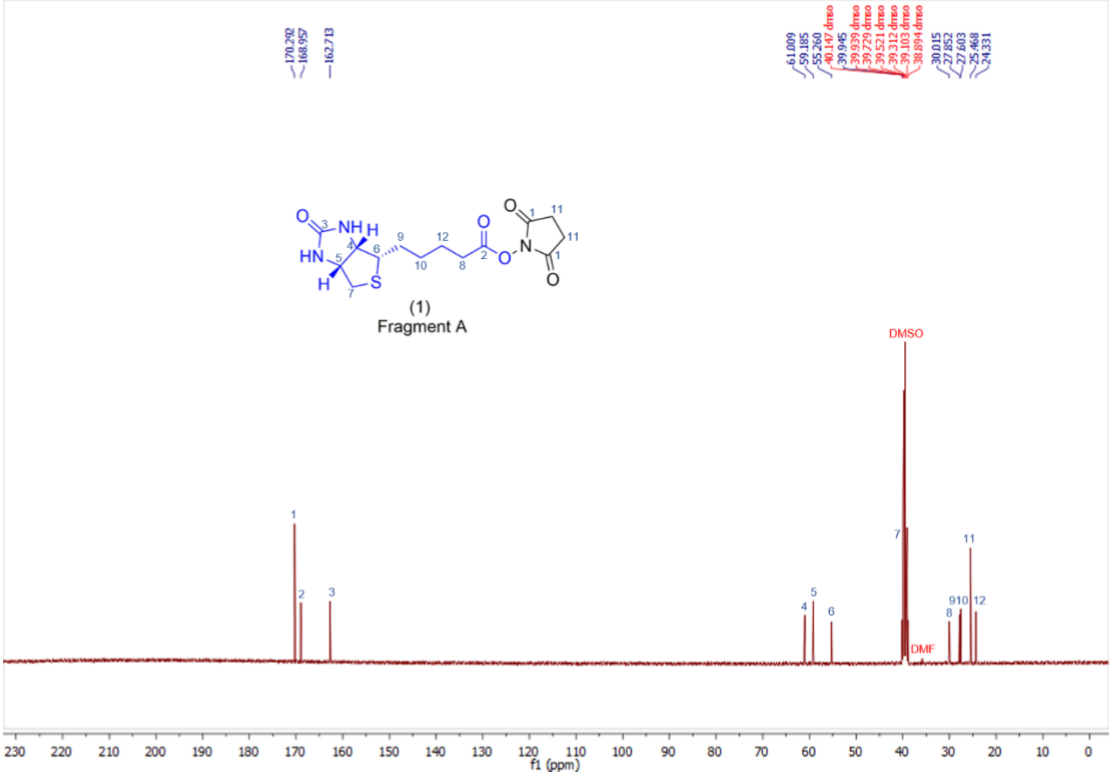

^{1}H NMR spectra for compound (**2**)

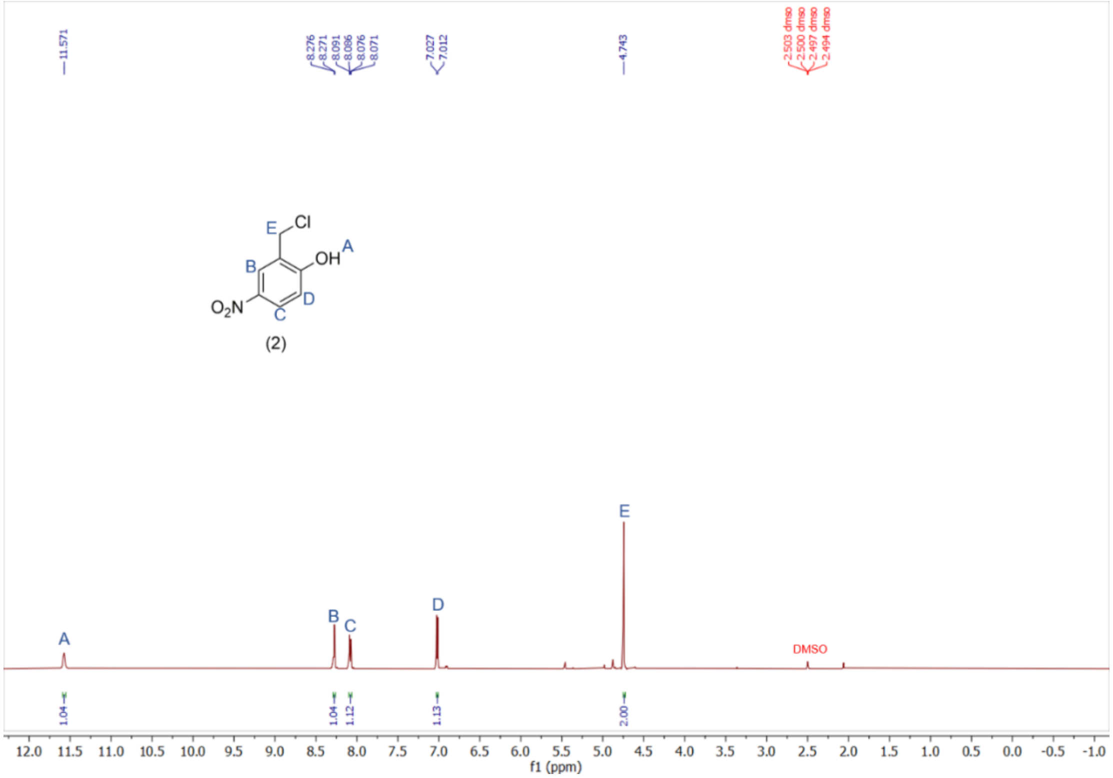

^{13}C NMR spectra for compound (**2**)

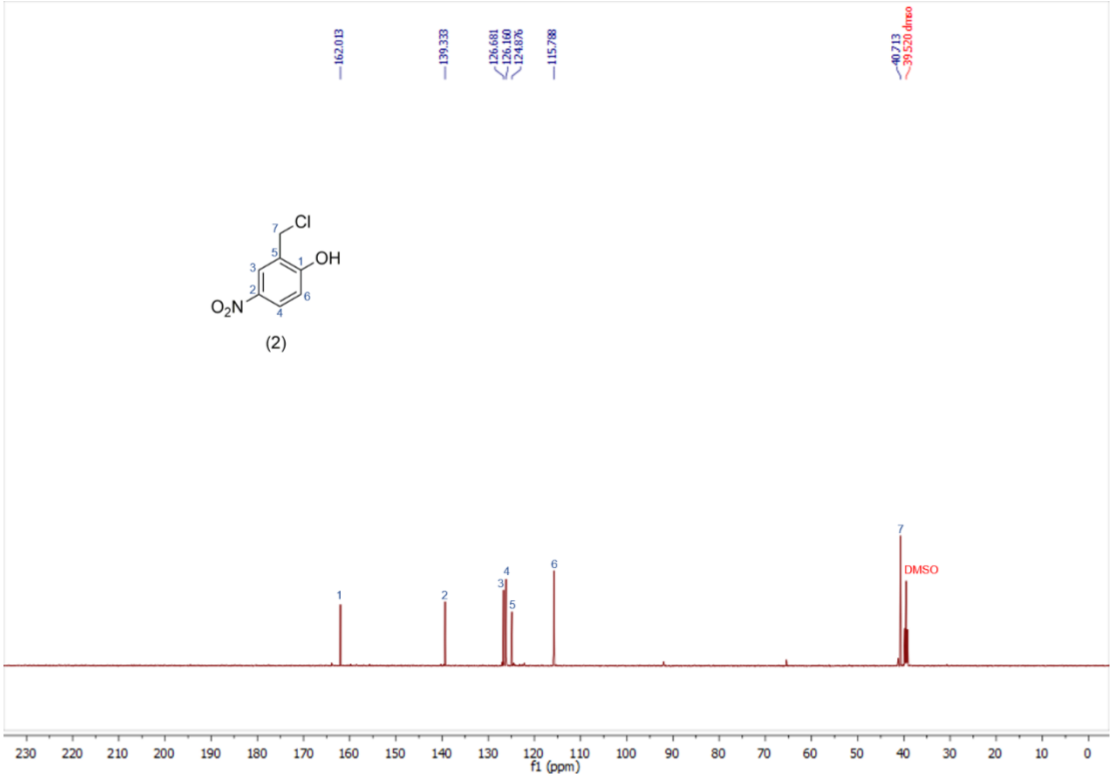

^{1}H NMR spectra for compound (**3**)

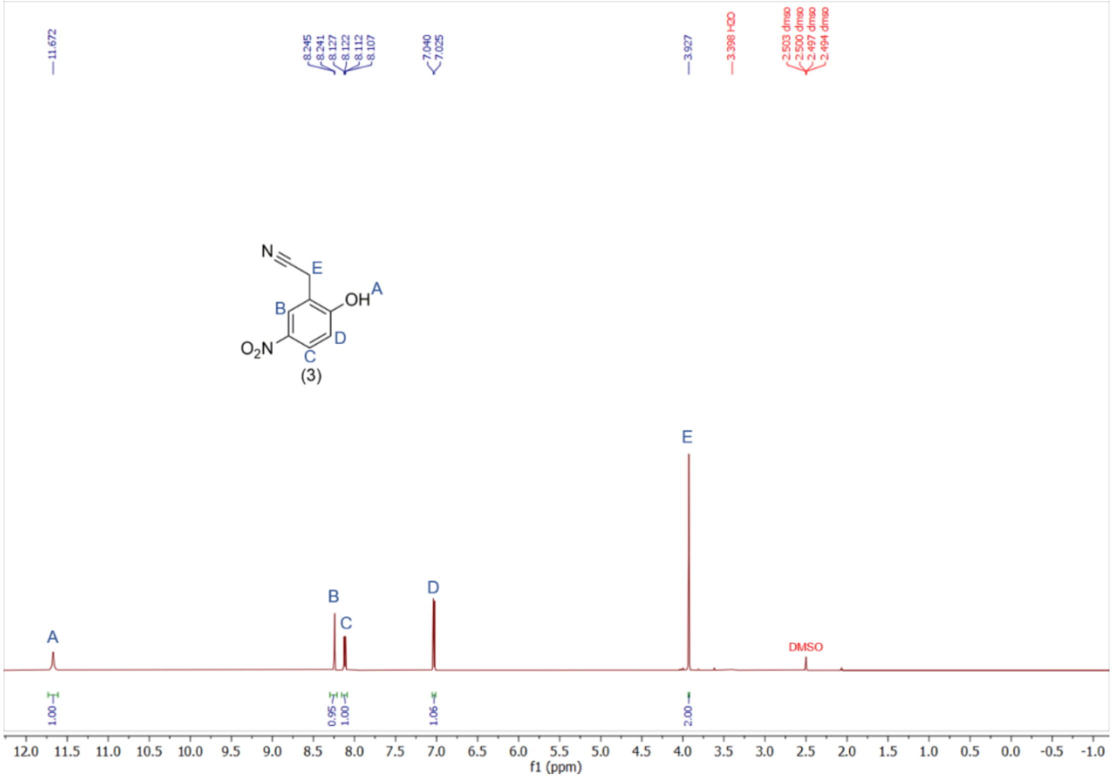

^{13}C NMR spectra for compound (**3**)

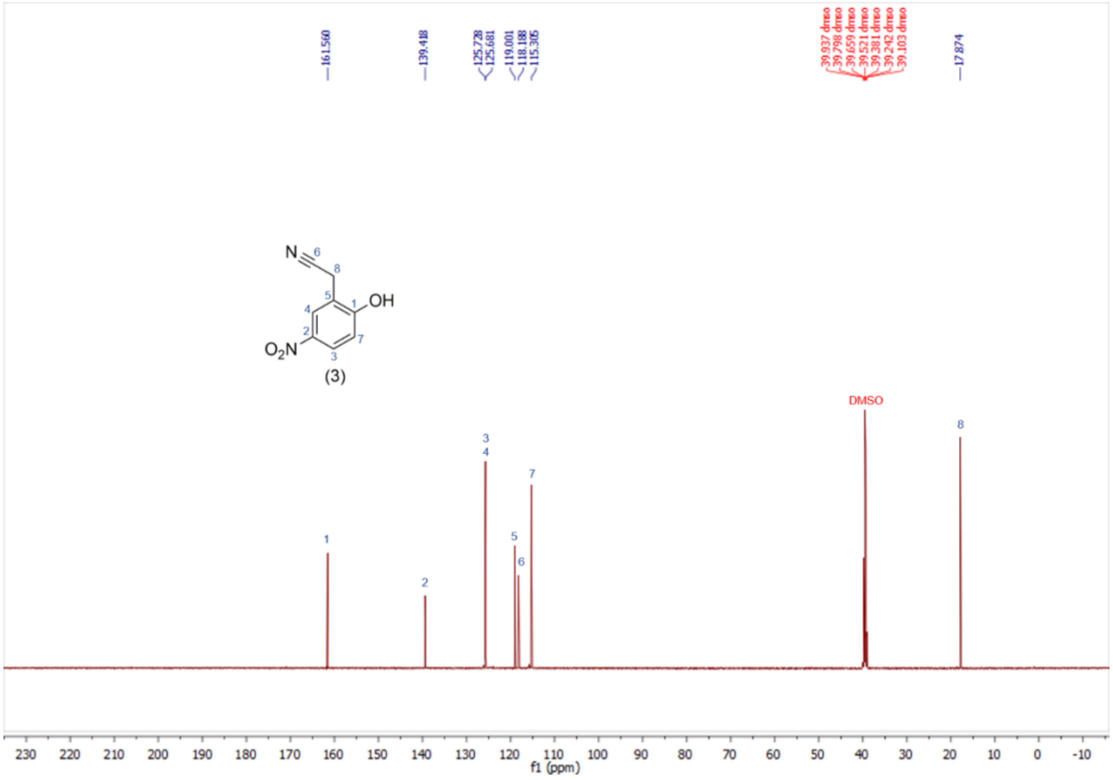

^{1}H NMR spectra for compound (**4**)

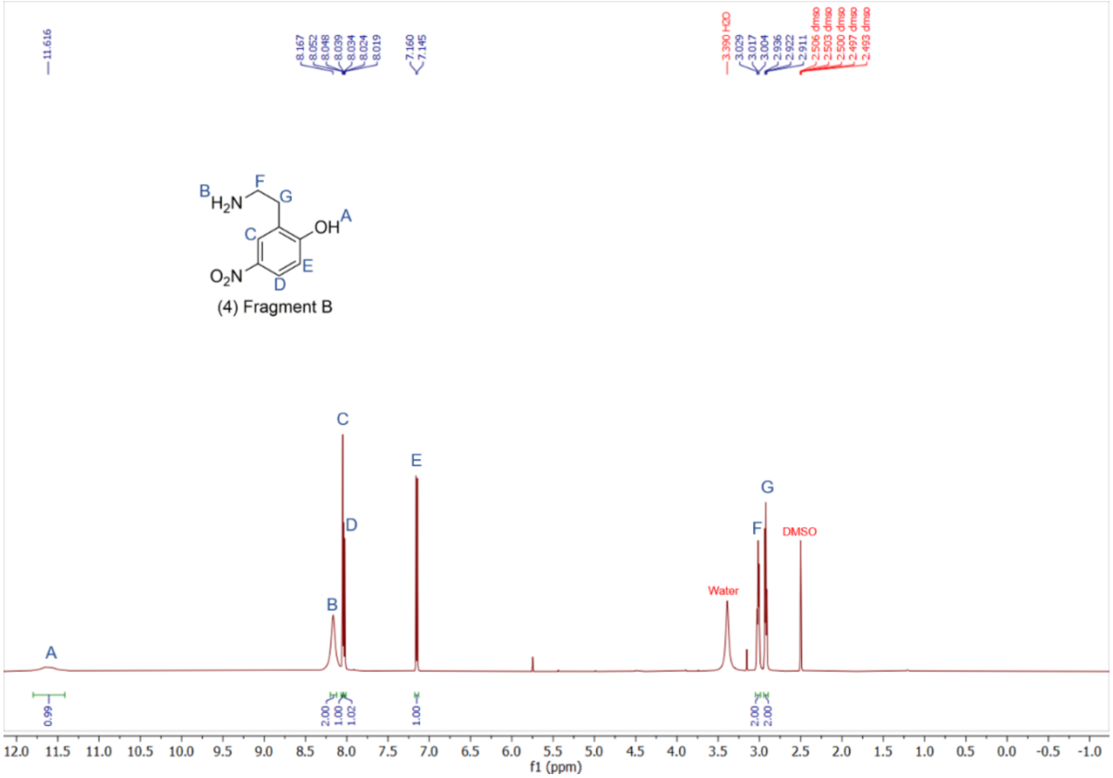

^{13}C NMR spectra for compound (**4**)

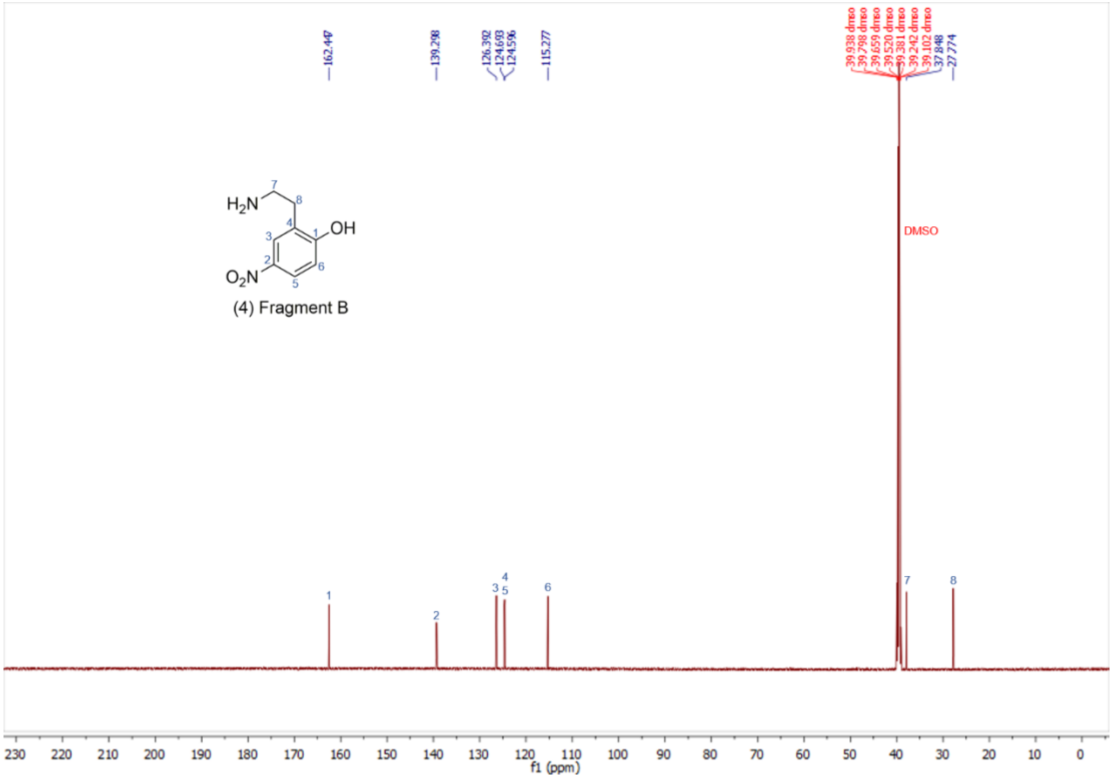

^{1}H NMR spectra for compound (**5**)

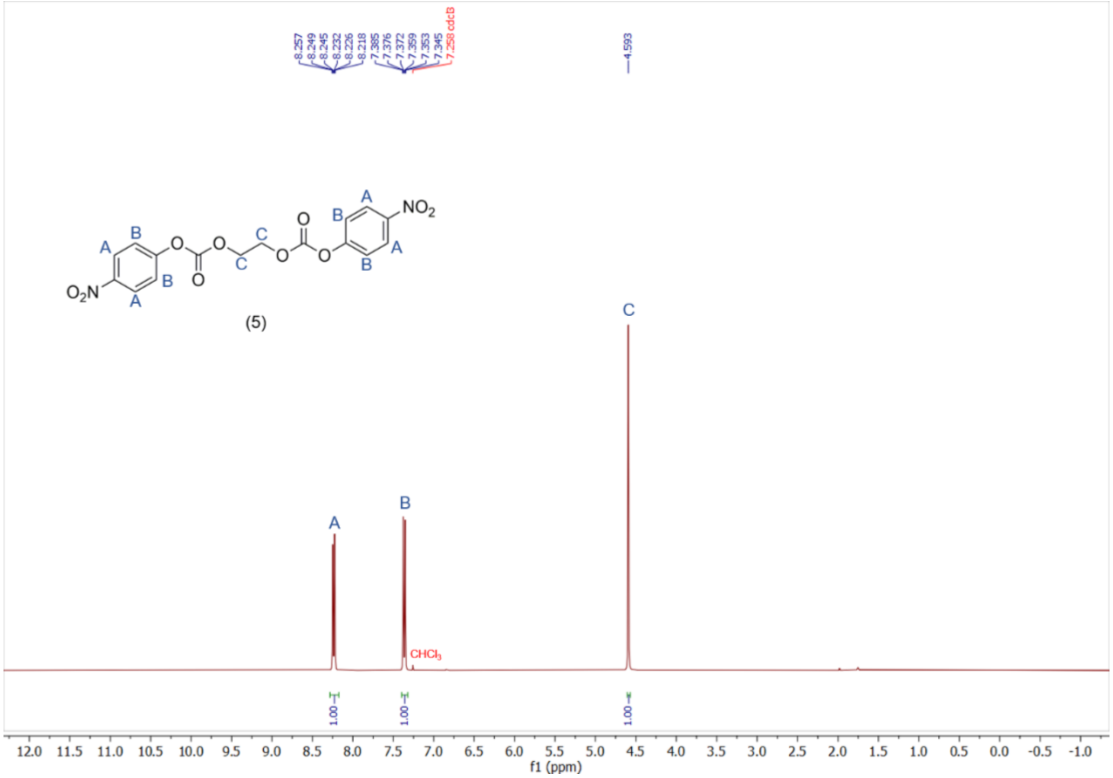

^{13}C NMR spectra for compound (**5**)

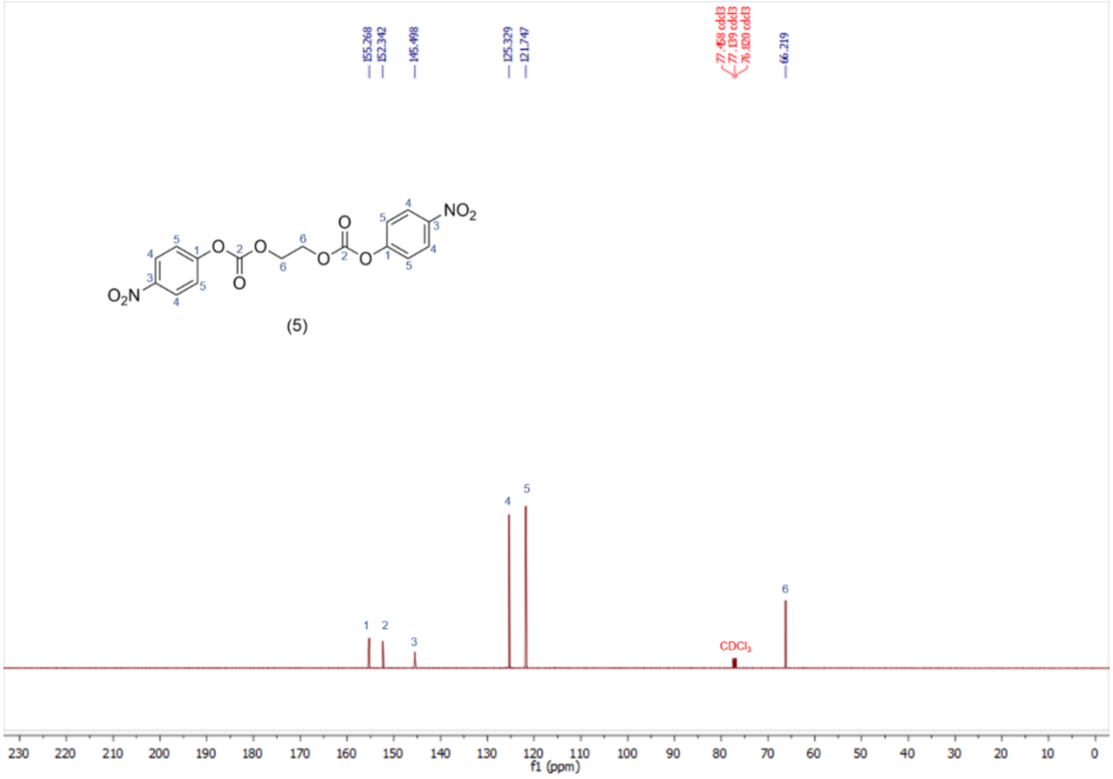

^{1}H NMR spectra for compound (**6**)

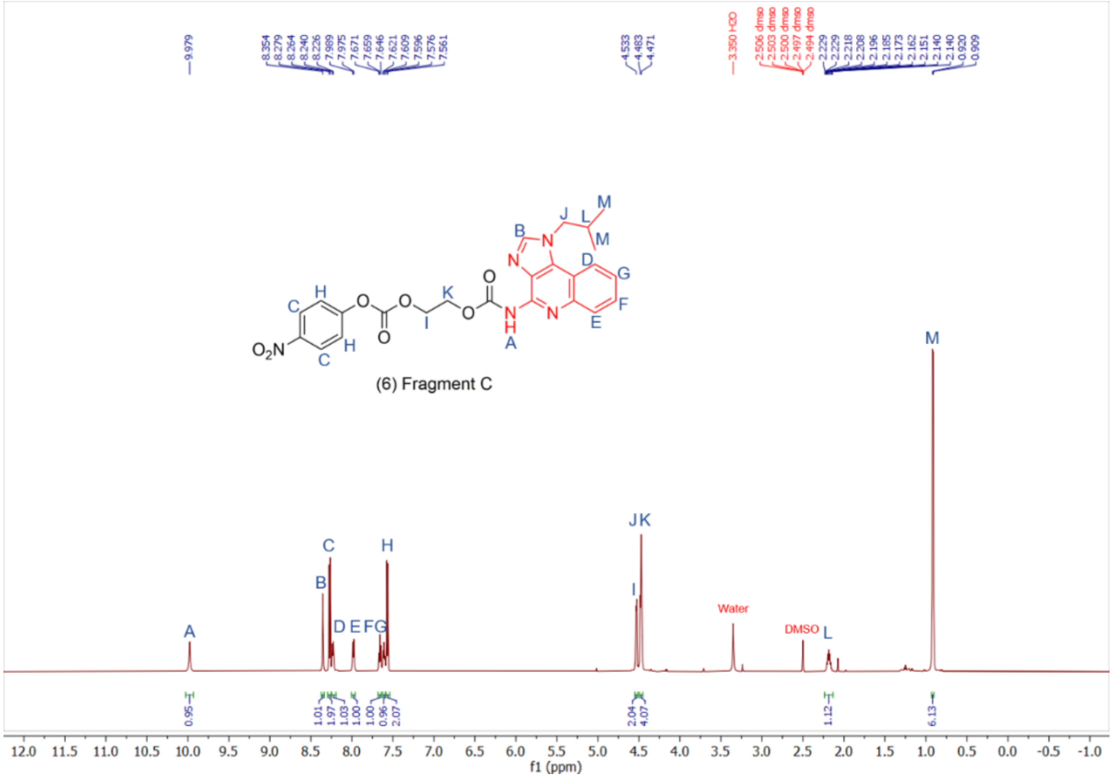

^{13}C NMR spectra for compound (**6**)

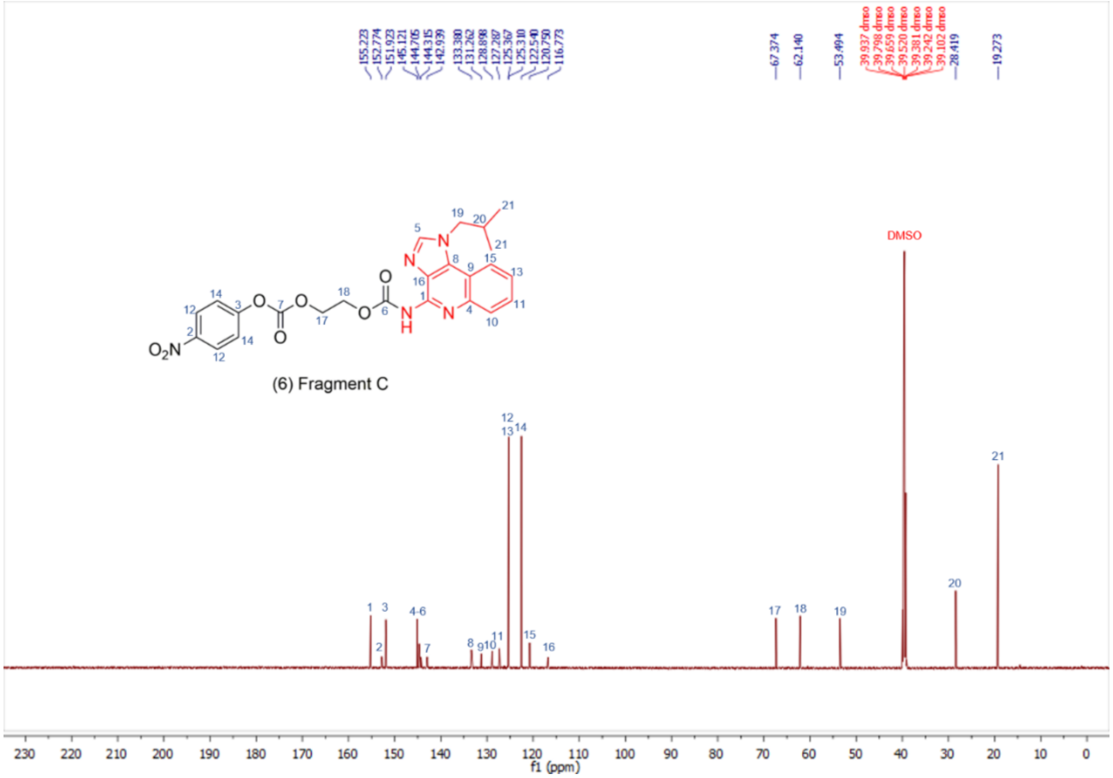

^{1}H NMR spectra for compound (**7**)

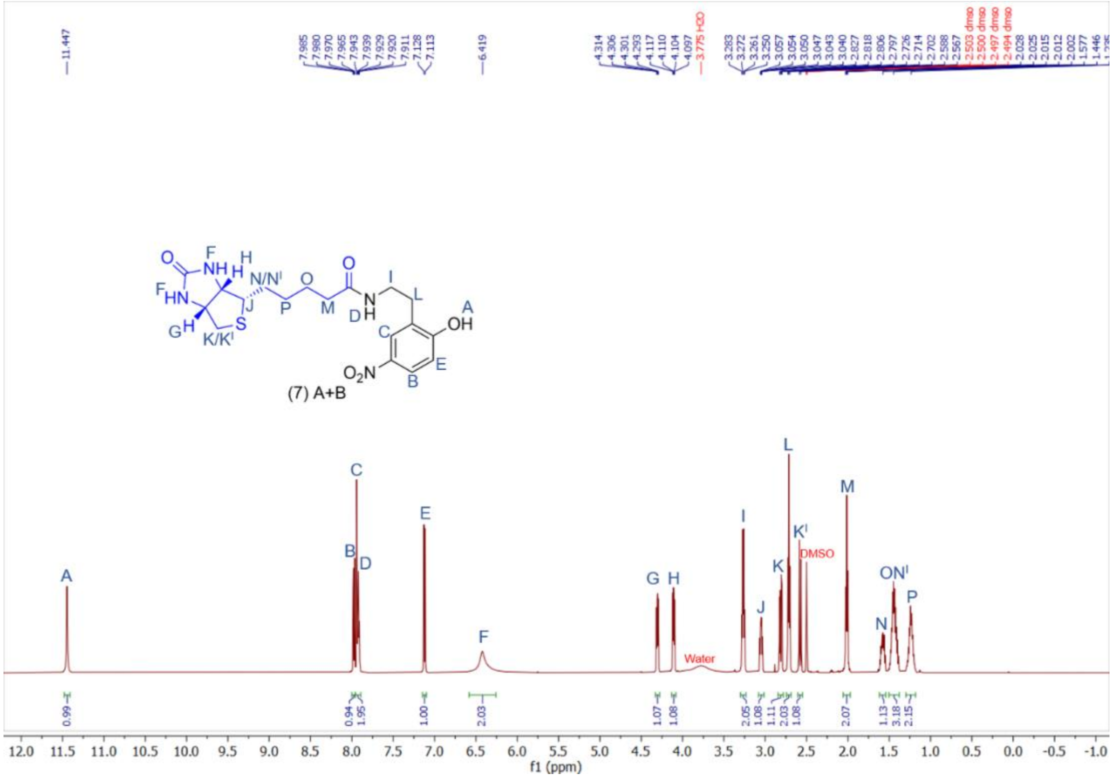

^{13}C NMR spectra for compound (**7**)

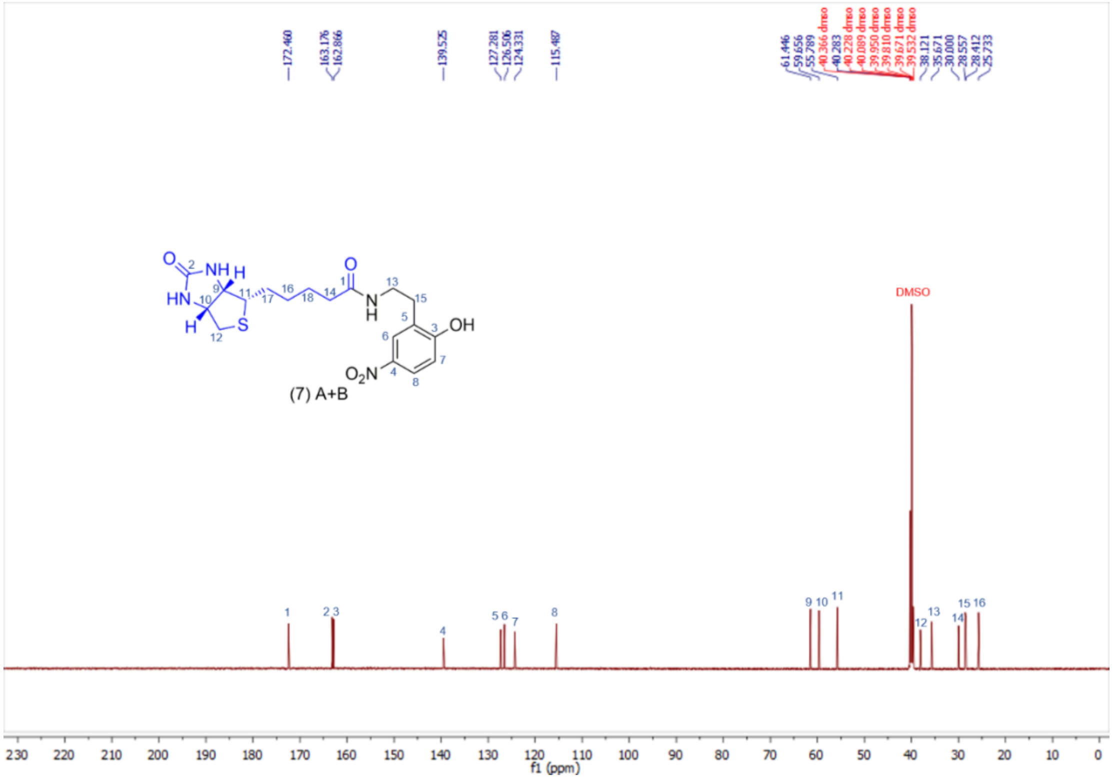

^{1}H NMR spectra for compound (**8**)

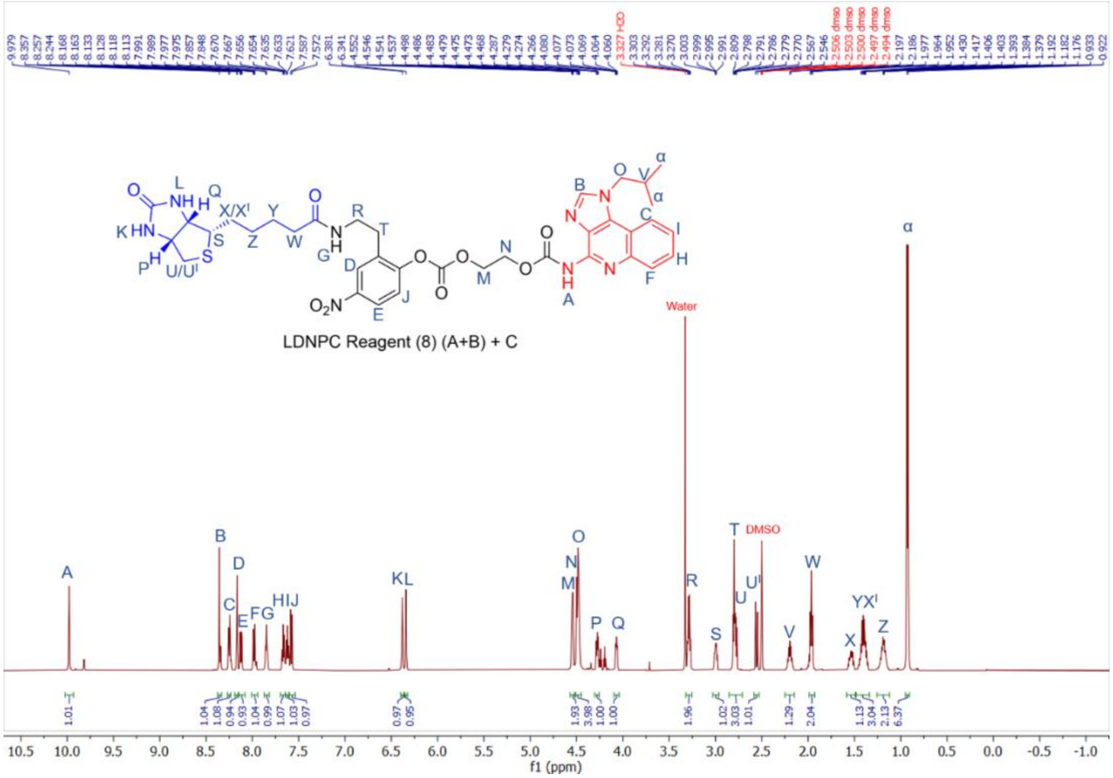

^{13}C NMR spectra for compound (**8**)

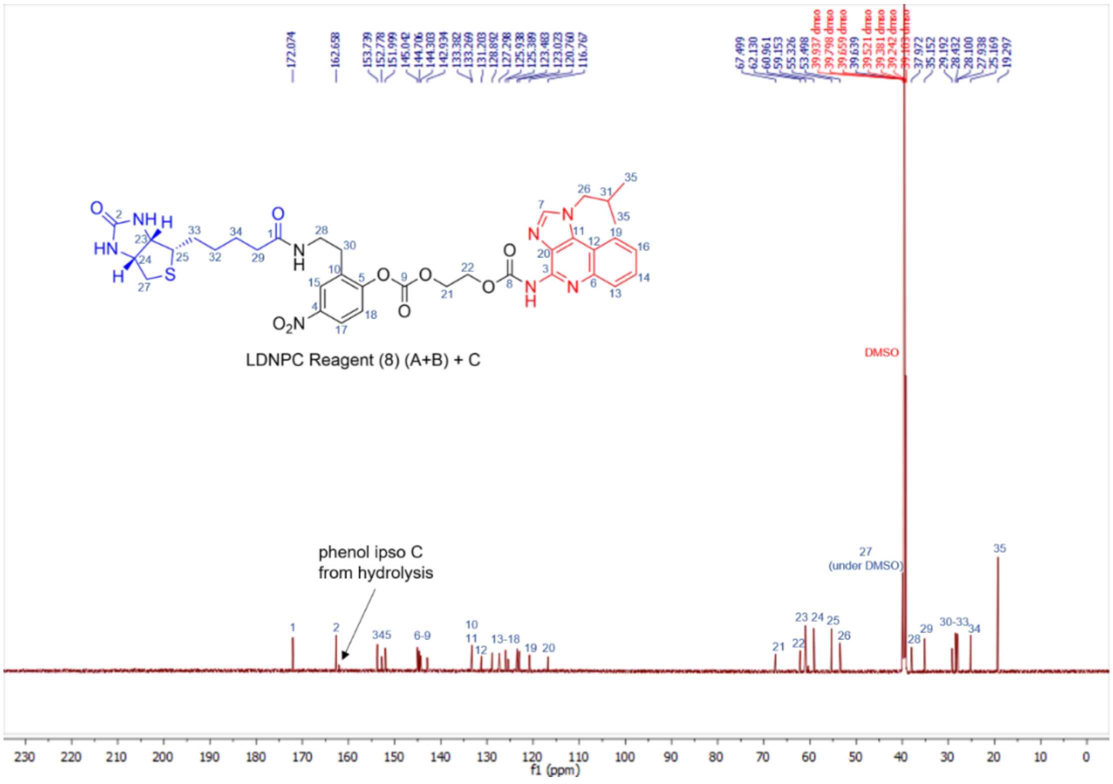

COSY NMR spectra for compound (**8**)

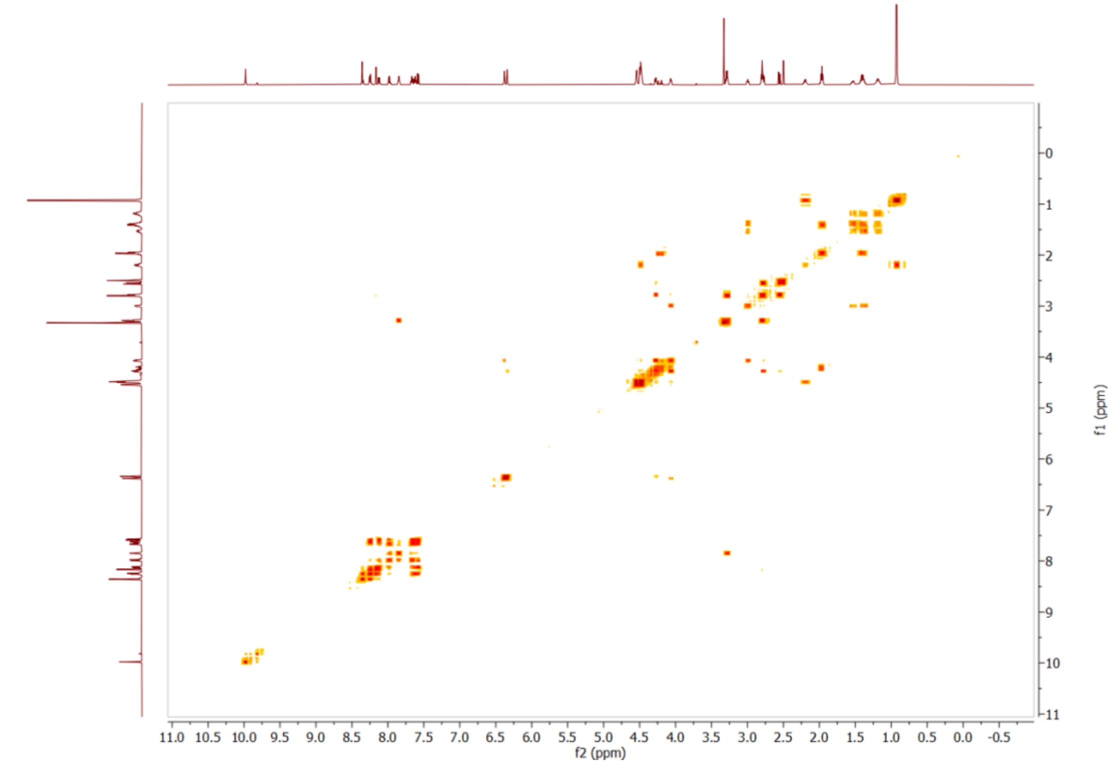

HSQC NMR spectra for compound (**8**)

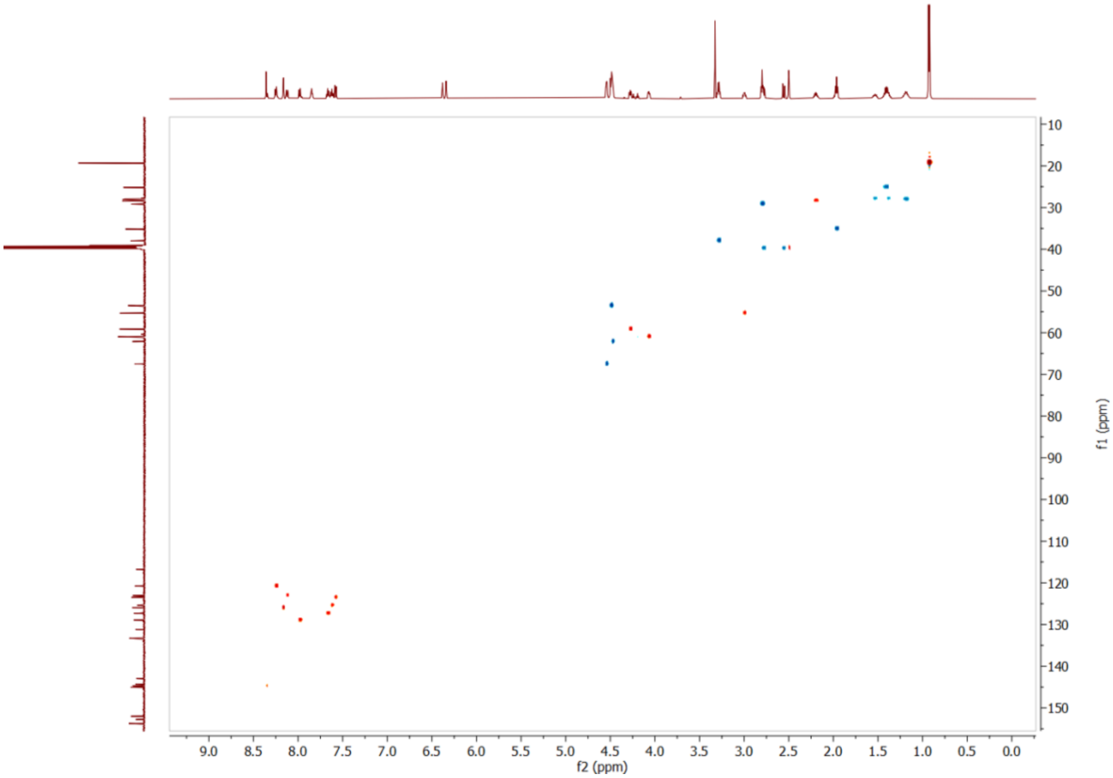

References:

[1] C. A. Buehler, F. K. Kirchner, G. F. Deebel, *Org. Synth.* **1940**, *20*, 59.

[2] International Union of Pure and Applied Chemistry, E. P. Serjeant, B. Dempsey, International Union of Pure and Applied Chemistry, Eds. , *Ionisation Constants of Organic Acids in Aqueous Solution*, Pergamon Press, Oxford ; New York, **1979**.

[3] Z. Otwinowski, W. Minor, in *Methods Enzymol.*, Elsevier, **1997**, pp. 307–326.

[4] P. D. Adams, P. V. Afonine, G. Bunkóczi, V. B. Chen, I. W. Davis, N. Echols, J. J. Headd, L.-W. Hung, G. J. Kapral, R. W. Grosse-Kunstleve, A. J. McCoy, N. W. Moriarty, R. Oeffner, R. J. Read, D. C. Richardson, J. S. Richardson, T. C. Terwilliger, P. H. Zwart, *Acta Crystallogr. D Biol. Crystallogr.* **2010**, *66*, 213–221.

[5] P. Emsley, B. Lohkamp, W. G. Scott, K. Cowtan, *Acta Crystallogr. D Biol. Crystallogr.* **2010**, *66*, 486–501.

[6] C. Yung-Chi, W. H. Prusoff, *Biochem. Pharmacol.* **1973**, *22*, 3099–3108.
